# Mitochondrial phylogenomics of the Bivalvia (Mollusca): searching for the origin and mitogenomic correlates of doubly uniparental inheritance of mtDNA

**DOI:** 10.1186/1471-2148-10-50

**Published:** 2010-02-18

**Authors:** Hélène Doucet-Beaupré, Sophie Breton, Eric G Chapman, Pierre U Blier, Arthur E Bogan, Donald T Stewart, Walter R Hoeh

**Affiliations:** 1 Département de Biologie, Université du Québec à Rimouski, 300 Allée des Ursulines, Rimouski, Québec, G5L 3A1, Canada; 2 Department of Biological Sciences, Kent State University, Kent, Ohio 44242, USA; 3 Department of Entomology, University of Kentucky, Lexington, Kentucky, 40546-0091 USA; 4 North Carolina State Museum of Natural Sciences, Research Laboratory, MSC 1626, Raleigh, North Carolina, 27699-1626 USA; 5 Department of Biology, Acadia University, 33 Westwood Ave, Wolfville, NS, B4P 2R6, Canada

## Abstract

**Background:**

Doubly uniparental inheritance (DUI) is an atypical system of animal mtDNA inheritance found only in some bivalves. Under DUI, maternally (F genome) and paternally (M genome) transmitted mtDNAs yield two distinct gender-associated mtDNA lineages. The oldest distinct M and F genomes are found in freshwater mussels (order Unionoida). Comparative analyses of unionoid mitochondrial genomes and a robust phylogenetic framework are necessary to elucidate the origin, function and molecular evolutionary consequences of DUI. Herein, F and M genomes from three unionoid species, *Venustaconcha ellipsiformis, Pyganodon grandis *and *Quadrula quadrula *have been sequenced. Comparative genomic analyses were carried out on these six genomes along with two F and one M unionoid genomes from GenBank (F and M genomes of *Inversidens japanensis *and F genome of *Lampsilis ornata*).

**Results:**

Compared to their unionoid F counterparts, the M genomes contain some unique features including a novel localization of the *trnH *gene, an inversion of the *atp8-trnD *genes and a unique 3'coding extension of the cytochrome *c *oxidase subunit II gene. One or more of these unique M genome features could be causally associated with paternal transmission. Unionoid bivalves are characterized by extreme intraspecific sequence divergences between gender-associated mtDNAs with an average of 50% for *V. ellipsiformis*, 50% for *I. japanensis*, 51% for *P. grandis *and 52% for *Q. quadrula *(uncorrected amino acid p-distances). Phylogenetic analyses of 12 protein-coding genes from 29 bivalve and five outgroup mt genomes robustly indicate bivalve monophyly and the following branching order within the autolamellibranch bivalves: ((Pteriomorphia, Veneroida) Unionoida).

**Conclusion:**

The basal nature of the Unionoida within the autolamellibranch bivalves and the previously hypothesized single origin of DUI suggest that (1) DUI arose in the ancestral autolamellibranch bivalve lineage and was subsequently lost in multiple descendant lineages and (2) the mitochondrial genome characteristics observed in unionoid bivalves could more closely resemble the DUI ancestral condition. Descriptions and comparisons presented in this paper are fundamental to a more complete understanding regarding the origins and consequences of DUI.

## Background

Mitochondrial DNA (mtDNA) is the only extranuclear genome in animal cytoplasm. Located in the matrix of mitochondria, metazoan mtDNA is normally a small circular DNA molecule about 14-16 kilobases (kb) long usually encoding the same 37 genes ([1,2]; but see [3] for exceptions). Typically, all mtDNAs in the zygote come from the oocyte and even though evidence for occasional paternal leakage has been reported [4,5], animal mtDNA is thought to strictly follow maternal inheritance [6]. This clonal inheritance coupled with the successive cell divisions that represent sequential bottlenecks for the mitochondrial population [6-8] result in an essentially homoplasmic state for mtDNA. An extreme exception to the paradigm of strict maternal inheritance of animal mtDNA (SMI) is found in three bivalve lineages (i.e., the orders Mytiloida, Unionoida and Veneroida), which possess an unusual system termed doubly uniparental inheritance of mtDNA (DUI) (see [9,10] for reviews).

In DUI-possessing organisms, distinct gender-associated mitochondrial DNA lineages coexist: a female-transmitted (F) genome and a male-transmitted (M) genome. Under DUI, female bivalves transmit their mitochondria (carrying F mtDNA) to both sons and daughters, as in SMI, but males pass on their mitochondria (via sperm carrying M mtDNA) to only sons (e.g., [11] but see [12]). At the organismal level, male bivalves with DUI are thus heteroplasmic and contain both M and F genomes. In male somatic tissues, the F genome predominates while in male gonadal tissues, the M genome is predominant [13] and it appears to be the exclusive type in sperm [14]. In females, both somatic and gonadal tissues typically contain the F genome, but the occasional presence of a small amount of the M genome has been demonstrated in somatic tissues and ovaries of some species [12-16].

The broad taxonomic distribution of DUI within the Bivalvia (e.g., [17-26]) reinforces the idea that it evolved once in an ancestral bivalve lineage, from standard uniparental inheritance, and was lost in some descendant bivalve lineages (e.g., oysters and probably scallops) [23,27,28]. DUI could then be the ancestral condition for the Bivalvia, however, a more definitive statement to this effect rests on producing a more reliable bivalve phylogeny along with clarifying the distribution of DUI in additional bivalve lineages. Although many of the essential elements of DUI have been described, (i.e., distinct M and F lineages, heteroplasmy in males, rapid molecular evolution particularly of M types [17-19,21,22,24,29-31], the current and/or historical function of DUI still remains a mystery. Comparisons of entire F and M genomes (as opposed to partial sequences of a few genes) will enable the characterization of potential gene content/organizational/functional differences between the M and F genomes, and will help to reconstruct the history of any possible recombination and/or gene translocation events in these distinct, gender-associated lineages.

To date, 15 complete or nearly complete F and M mtDNA genome sequences are available for species with DUI but these are numerically biased towards marine taxa (i.e., species from the Mytiloida and Veneroida) [32-37] (Table 1). While the vertebrate mitochondrial gene order is almost invariant, mollusks, and bivalves in particular, exhibit radical rearrangements of mitochondrial genes and extensive mtDNA variability at the intrageneric level [2,38,39]. For example, the two congeneric oyster species *Crassostrea virginica *and *C. gigas*, both lacking DUI [28], show broad differences in gene content and gene order with relocation of most tRNA genes [2,40]. At the species level, the extent of genome rearrangement between the two distinct gender-associated mitochondrial genomes appears to vary greatly among the three divergent bivalve lineages. In the Mytiloida, the gene order and content of F and M genomes from a species are conserved but both lack the gene for ATPase subunit 8 (*atp8*) and have a second tRNA gene for methionine (*trnM*) [31,34,36]. By contrast, in the marine clam *Venerupis philippinarum*, gene content differs between M and F genomes as we observe a gene duplication for the cytochrome *c *oxidase subunit II gene (*cox2*) in the F genome and an extra *trnM *in the M genome, and both genomes have a short *atp8 *gene (37 amino acids) the function of which is unclear [2,41]. In freshwater mussels, an M genome-specific 3' extension of the cytochrome *c *oxidase subunit II gene (M*cox2*) has been clearly demonstrated [22,25,42]. This functional extension is a unique feature of unionoid M genomes and typically yields an ~80% increase in gene length relative to the female-transmitted *cox2 *gene [43]. In unionoid bivalves, the presence of *atp8 *has been confirmed in both M and F genomes [2,37]. Also, the F genomes of *Inversidens japanensis *and *Hyriopsis cumingii *(both in the subfamily Gonideinae) exhibit a different gene order compared with the M genome of *I. japanensis *and the F genome of *Lampsilis ornata *([37]; Zheng RL and Li JL, personal communication) (see Table 1). Analysis of gene order for additional F and M mtDNA genomes from freshwater mussels will allow us to test the following alternative hypotheses: (1) the translocation of several genes as observed in the F genomes of *I. japanensis *and *H. cumingii *represents an idiosyncratic gene rearrangement unique to these species or to the subfamily Gonideinae or (2) that this gene arrangement is in fact a characteristic shared with other unionoid species' F genomes or other subfamily.

**Table 1:** Species and GenBank accession numbers of the sequences used in this study for phylogenetic analyses.

Species	Gender	GenBank accession Numbers
Bivalvia		
Autolamellibranchiata		
Unionoida		
*Venustaconcha ellipsiformis*	F	FJ809753
*Venustaconcha ellipsiformis*	M	FJ809752
*Pyganodon grandis*	F	FJ809754
*Pyganodon grandis*	M	FJ809755
*Inversidens japanensis*	F	AB055625
*Inversidens japanensis*	M	AB055624
*Quadrula quadrula*	F	FJ809750
*Quadrula quadrula*	M	FJ809751
*Lampsilis ornata*	F	NC_005335
*Hyriopsis cumingii*	F	NC_011763
Veneroida		
*Venerupis philippinarum*	F	NC_003354
*Venerupis philippinarum*	M	AB065374
*Acanthocardia tuberculata*	-	NC_008452
*Sinonovacula constricta*	-	NC_011075
*Hiatella arctica*	-	NC_008451
Pteriomorphia		
Mytiloida		
*Mytilus trossulus*	F	DQ198231
*Mytilus trossulus*	M	DQ198225
*Mytilus edulis*	F	NC_006161
*Mytilus edulis*	M	AY823624
*Mytilus galloprovincialis*	F	NC_006886
*Mytilus galloprovincialis*	M	AY363687
Ostreoida		
*Crassostrea hongkongensis*	-	NC_011518
*Crassostrea gigas*	-	NC_001276
*Crassostrea virginica*	-	NC_007175
Pectinoida	-	NC_009687
*Argopecten irradians*		
*Placopecten magellanicus*	-	NC_007234
*Mizuhopecten yessoensis*	-	NC_009081
*Chlamys farreri*	-	NC_012138
*Mimachlamys nobilis*	-	NC_011608
**Out-group**		
*Aplysia californica *(Gastropoda)	-	NC_005827
*Graptacme eborea *(Scaphopoda)	-	NC_006162
*Katharina tunicata *(Polyplacophora)	-	NC_001636
*Octopus vulgaris *(Cephalopoda)	-	NC_006353
*Platynereis dumerii *(Polychaeta)	-	NC_000931

Having additional unionoid F and M genomes available for comparative analyses would also significantly illuminate investigations into the likely unique origin of DUI [26]. Analyses of morphological and molecular datasets indicate that unionoid bivalves, together with trigonioid bivalves, compose a monophyletic subclass, the Paleoheterodonta, [44-49]. The relative antiquity of this subclass within the Bivalvia is supported by the molecular sequence-based phylogenies, presented in Hoeh et al. ([45]: Figure three), Giribet and Wheeler ([47]: Figure five), Giribet and Distel ([48]: Figure three point five) and Dreyer and Steiner ([41]: Figure five), which suggest that the Paleoheterodonta are a product of an early cladogenic event in extant autolamellibranch (~suspension-feeding) bivalves. Given the hypothesized relatively basal position of unionoids in the bivalve phylogeny, their mt genomes could retain ancestral character states that are informative with respect to the initial mt genome duplication event (i.e., the formation of a distinct male-transmitted lineage in addition to a female-transmitted lineage) that led to the evolution of DUI.

In the present study, six new complete mitochondrial genomes, namely, the F and M genomes of the unionoid bivalves *Venustaconcha ellipsiformis *(Unionoida: Unionidae: Ambleminae: Lampsilini), *Pyganodon grandis *(Unionoida: Unionidae: Unioninae: Anodontini) and *Quadrula quadrula *(Unionoida: Unionidae: Ambleminae: Quadrulini), were compared with the available complete genomes of DUI species deposited in GenBank and their gene order, gene content and variation were analyzed. Additionally, complete bivalve mt genomes were phylogenetically analyzed to further test the hypothesized basal position of the Paleoheterodonta among extant autolamellibranch bivalves and to evaluate the evolutionary history of mitogenomic character state changes. The aim is to provide a context for comparisons of mt genomes among DUI and non-DUI bivalve lineages, and ultimately to identify the gene region(s) involved in the manifestation of DUI. Such descriptions and comparisons will contribute to a more complete picture of the evolution not only of the DUI system *per se*, but also of the factors involved in the near universal presence of SMI in animals.

## Results

### Phylogenetic analysis

**Figure 1:**
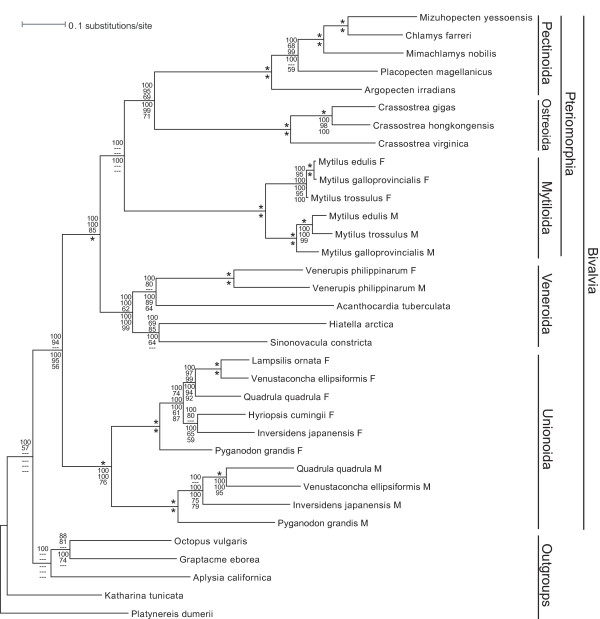
**Bayesian inference majority-rule tree of bivalve mt genome relationships based on an analysis using the M3 codon substitution model and a nucleotide alignment of 12 mitochondrial protein-coding genes (*atp8 *excluded)**. Numbers above an internal branch, from top to bottom, indicate nodal support values from BI, ML and MP nucleotide-based analyses, respectively. Numbers below an internal branch, from top to bottom, indicate nodal support values from BI, ML and MP amino acid-based analyses, respectively. Only nodal support values > 50% are presented. An asterisk above an internal branch indicates that all three nucleotide-based nodal support values are 100; an asterisk below an internal branch indicates that all three amino acid-based nodal support values are 100. Branch lengths reflect substitutions per site and the taxonomic and gender-specific transmission affiliations of the individual sequences are indicated at the right. All phylogenetic analyses strongly indicate that the unionoids represent the basal lineage for the bivalve taxa represented in this analysis.

**Figure 2:**
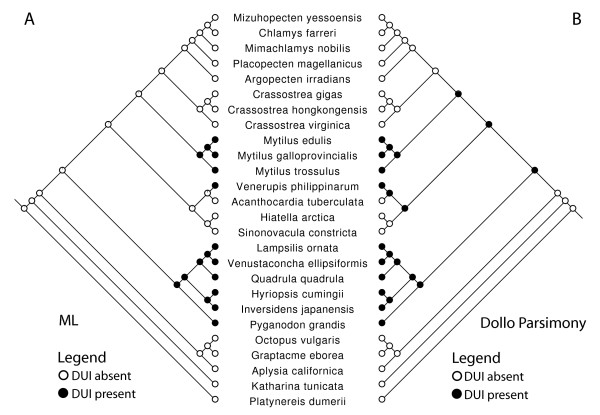
**ML-based (A) and Dollo parsimony-based (B) ancestral character state reconstructions of presence/absence of DUI on a species-level tree derived from the tree presented in Figure 1**. The ML-based reconstruction indicates three separate origins of DUI while the Dollo parsimony-based reconstruction indicates a single origin of DUI followed by three reversals to SMI.

The majority-rule codon-based BI tree (Figure 1), derived from using concatenated sequences of mitochondrial protein-coding genes, is well resolved and very similar in topology to the best BI tree produced from analysis of amino acids as well as to the best nucleotide- and amino acid-based ML and parsimony trees (not shown). In summation, these trees clearly indicate that (1) the Bivalvia is monophyletic, (2) pteriomorph and veneroid bivalves are reciprocally monophyletic with unionoid bivalves being sister to Pteriomorphia+Veneroida, (3) the F and M clades in unionoids are reciprocally monophyletic and (4) branch lengths indicate the higher substitution rate of the unionoid M genome relative to that of the unionoid F genome. The ML reconstruction of the presence/absence of DUI (Figure 2A), using the MK1 model, unambiguously indicates three origins of DUI for the taxa included in this study.

### Genome structural features

**Figure 3:**
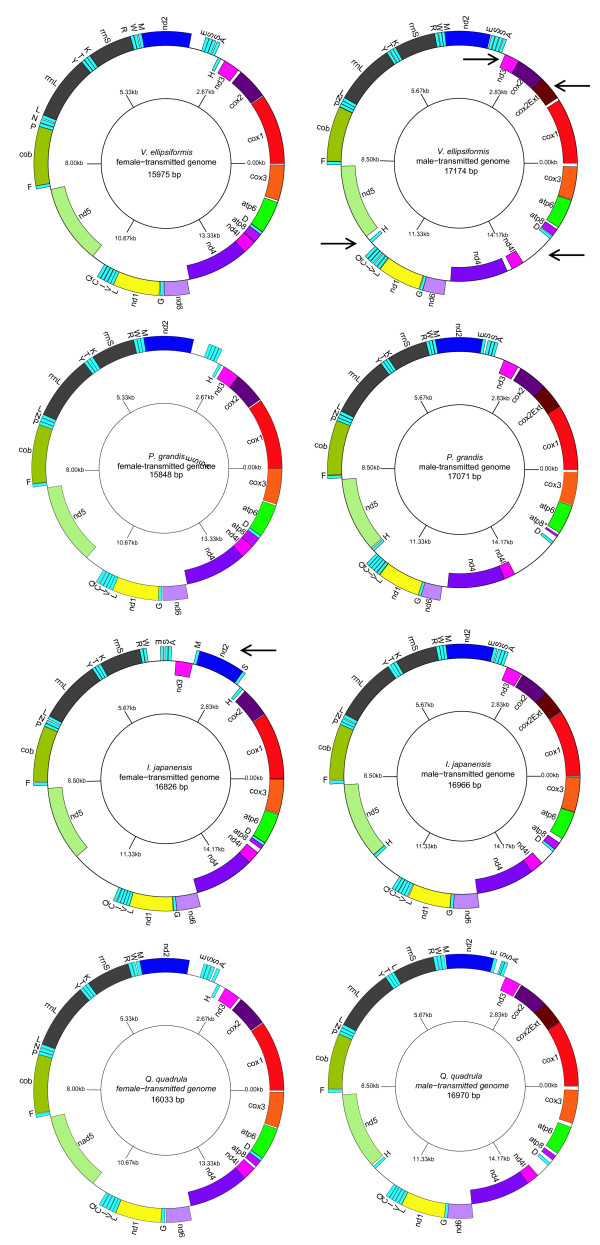
**Gene maps of the M and F mitochondrial genomes of *Venustaconcha ellipsiformis, Pyganodon grandis, Inversidens japanensis *and *Quadrula quadrula***. Protein and rRNA genes are named as in the text while tRNA genes are abbreviated by the one-letter code of the corresponding amino acid (L1 = *trnL *(*cua*), L2 = *trnL *(*uaa*), S1 = *trnS *(*aga*), and S2 = *trnS *(*uaa*)). Genes positioned inside the plain line are encoded on the heavy strand and genes outside the line are encoded on the light strand. Atp8* (= genomes lacking full size *atp8 *gene). Black arrows on the *V. ellipsiformis *M genome indicate regions that differ between male- and female-transmitted genomes and the arrow on the *I. japanensis *F genome indicates the region with a gene order distinct from that of the other figured F genomes. The circular gene maps of the genomes were drawn by GenomeVx [113] followed by manual modification.

The length of the F *V. ellipsiformis*, F *P. grandis *and F *Q. quadrula *mitochondrial genomes are 15,975 bp, 15,848 bp and 16,033 bp, respectively. These sizes are ~950 bp to 1,200 bp smaller than their M counterparts whose genomes are 17,174 bp (*V. ellipsiformis*), 17,071 bp (*P. grandis*) and 16,970 bp (*Q. quadrula*). The length differences are mainly due to the presence of a unique M genome-specific 3' extension of the cytochrome *c *oxidase subunit 2 (*cox2*) gene and longer unassigned/noncoding regions in M genomes. The A+T composition is very similar among the six newly sequenced genomes but higher than F and M *I. japanensis *(Table 2). Ribosomal RNA genes and protein-coding genes (except *atp8*) of M and F genomes of *V. ellipsiformis, P. grandis *and *Q. quadrula *are arranged identically but tRNA order differs among the analyzed genomes (Figure 3). In unionoids, ten or eleven genes out of ~37 are located on one strand and all the other genes on the opposite (Figure 3).

**Table 2:** Main structural features of the female- and male-transmitted mitochondrial genomes of *Venustaconcha ellipsiformis, Pyganodon grandis, Inversidens japanensis *and *Quadrula quadrula*.

	***V***. *ellipsiformis* Female	***V***. *ellipsiformis* Male	*P. grandis* Female	*P. grandis* Male	*I. japanensis* Female	*I. japanensis* Male	*Q. quadrula* Female	*Q. quadrula* Male
**Total size**	15 975	17 174	15 848	17 071	16 826	16 966	16 033	16 970
**pb**								
**A+T %**	62.55	63.47	64.22	64.76	57.20	57.12	61.94	61.80
**Strand - CG-skew**	-0.46	-0.49	-0.36	-0.33	-0.42	-0.41	-0.48	-0.51
**Strand - AT-skew**	-0.40	-0.41	-0.30	-0.25	-0.41	-0.41	-0.44	-0.43
**Strand + CG-skew**	0.32	0.31	0.28	0.25	0.26	0.30	0.33	0.38
**Strand + AT-skew**	-0.06	0.04	-0.09	-0.08	0.00	0.05	-0.02	0.10
***rrnS***	865	859	847	841	844	864	857	865
***rrnL***	1271	1290	1271	1287	1304	1325	1297	1306
***cox1***	1542 (TTG/TAG)	1537 (GTG/T**)	1539 (TTG/TAA)	1537 (CTG/T**)	1539 (GTG/TAA)	1543 (TTG/T**)	1542 (TTG/TAG)	1534 (GTG/T**)
***cox2***	684 (ATG/TAA)	1233 (ATG/TAA)	681 (ATG/TAG)	1251 (ATG/TAG)	681 (ATG/TAA)	1224 (ATG/TAG)	681 (ATG/TAG)	1230 (ATG/TAG)
***Nd3***	357 (ATG/TAG)	354 (ATG/TAA)	357 (ATG/TAA)	357 (TTG/TAA)	381 (ATG/TAA)	360 (ATG/TAG)	360 (ATA/TAA)	366 (ATT/TAA)
***Nd2***	972 (ATG/TAG)	957 (ATT/TAA)	966 (ATG/TAG)	972 (ATT/TAA)	960 (ATG/TAA)	981 (ATA/TAG)	966 (ATG/TAA)	990 (ATC/TAA)
***cob***	1156 (ATT/T**)	1158 (ATG/TAA)	1146 (ATC/TAA)	1149 (TTG/TAA)	1152 (ATT/TAA)	1152 (GTG/TAA)	1152 (ATC/TAA)	1149 (ATG/TAA)
***Nd5***	1731 (ATA/TAA)	1758 (ATA/TAA)	1737 (GTG/TAG)	1740 (ATA/TAA)	1704 (GTG/TAG)	1765 (TTA/T**)	1734 (ATG/TAA)	1770 (ATG/TAA)
***Nd1***	903 (ATC/TAA)	903 (ATA/TAA)	900 (ATA/TAA)	897 (GTG/TAA)	897 (ATC/TAG)	879 (ATA/TAA)	894 (ATA/TAA)	912 (ATG/TAA)
***Nd6***	489 (ATT/TAA)	456 (ATA/TAG)	489 (ATC/TAA)	405 (ATA/TAA)	489 (ATC/TAA)	528 (ATA/TAG)	498 (ATG/TAA)	522 (ATA/TAG)
***Nd4***	1347 (ATT/TAG)	1350 (ATG/TAG)	1344 (ATT/TAA)	1347 (GTG/TAA)	1374 (GTG/TAA)	1362 (ATT/TAG)	1332 (TTT/TAA)	1380 (ATG/TAA)
***Nd4l***	297 (GTG/TAG)	297 (TTG/TAA)	297 (GTG/TAG)	264 (ATA/TAG)	297 (GTG/TAA)	309 (ATA/TAG)	294 (GTG/TAG)	297 (ATG/TAG)
***atp8***	210 (GTG/TAA)	174 (GTG/TAG)	225 (ATG/TAA)	Remnant	Remnant 96 (GTG/TAG)	181 (ATG/T**)	159 (ATG/TAA)	123 (GTG/TAA)
***atp6***	708 (ATG/TAG)	696 (ATG/TAA)	714 (ATG/TAG)	690 (ATG/TAA)	708 (ATG/TAG)	663 (ATG/TAG)	708 (ATG/TAG)	678 (ATA/TAG)
***cox3***	780 (ATG/TAG)	774 (ATT/TAA)	780 (ATG/TAA)	772 (ATG/T**)	795 (ATA/TAA)	810 (GTG/TAA)	780 (ATG/TAG)	777 (ATT/TAA)

NOTE.-- Gene lengths are in bp. For each protein-coding gene, start and stop codons are presented in parentheses.

#### tRNA Histidine

In the three newly sequenced M genomes of this study, tRNA histidine (*trnH*) is positioned between *nd5 *and *nd1 *while in F genomes it is located between *nd2 *and *nd3 *(Figure 3). The location of the *trnH *gene in the *I. japanensis *M genome has previously been identified between *cox1 *and *cox2 *(Okazaki M and Ueshima R, personal communication). This location also corresponds to M*cox2e*. However, our reannotation of the *I. japanensis *M genome identifies *trnH *between *nd5 *and *nd1 *as in the three new M genomes. Examination of 41 entire mt genomes across the Mollusca allows us to group some classes of mollusks according to the position of *trnH*. For example, ***nd5-trnH-nd4 ***(encoded on the **heavy strand**) is the common organization in the Cephalopoda while *nd4-trnH-nd5 *(encoded on the light strand) and *cox2-trnG-trnH-****trnQ***-***trnL2***-***atp8 ***(encoded on both strands) are two most common arrangements found in the Gastropoda. Except for unionoids genomes, no common arrangement is found in bivalves and the arrangement of the *trnH*-containing region appears to be unique to each genus or species sequenced to date. *Nd2-trnR-trnH-nd4 *is found in the genus *Crassostrea *spp. (oysters) whereas the marine mussels *Mytilus *spp. possess the arrangement *nd2-trnR-trnW-trnA-trnS-trnH-trnP-nd3*. Because of the current uncertainty regarding molluscan phylogeny, a rigorous ancestral character state reconstruction is not possible. However, the arrangement observed in *Mytilus *spp. could be an inversion (+ strand reversion) of the ***nd3-trnH****-trnA-trnS1-trnS2-nd2 *observed in the unionoid F genomes. In the four unionoid M genomes, *trnH *is located between *nd5 *and *nd1 *and the only other molluscan species with a similar location for its *trnH *(i.e., *nd5-trnL****-trnH-nd1***) is the patellogastropod limpet *Lottia digitalis*. The gene order of the *L. digitalis *mt genome is the most divergent among all gastropod mtDNAs sequenced thus far [50].

#### Extension of the M cytochrome c oxidase subunit II gene

The three analyzed M genomes possess the unique 3' extension of the cytochrome *c *oxidase subunit II gene (M*cox2e*) [22]. In the three newly sequenced M genomes, the extension is 187 codons (*V. ellipsiformis *and *P. grandis*) or 186 codons (*Q. quadrula*) in length while the *I. japanensis *extension is slightly shorter with 181 codons.

#### Atp8 gene

As in the F genome of *L. ornata *[37] and the reannotated F and M genomes of *I. japanensis *[2], the newly sequenced F and M genomes contain the 13 protein-coding genes commonly found in other animal mtDNAs. Only the M genome of *P. grandis *appears to lack a complete *atp8*. In this species, a remnant of the *atp8 *gene that corresponds to the first 15 amino acids (MPQLSPVYWVSIFFL) of the protein, and that shows similarities with other *atp8 *genes sequenced in this study, has been identified between *trnD *and *atp6 *(Figure 3). Those 15 amino acids are followed by a complete stop codon. After the stop codon, we also identified an open reading frame, in a different frame than the first 15 amino acids, which could correspond to the remainder of *atp8*.

#### NADH dehydrogenase subunits 4 and 4L genes

Most unionoid mt genomes examined in this study have an overlap of 7 bp for subunits 4 and 4L of the NADH dehydrogenase complex (*nd4 *and *nd4l*). Two exceptions are the M genome of *V. ellipsiformis*, which contains a noncoding region of 120 bp between *nd4 *and *nd4l*, and the F genome of *I. japanensis*, which possesses one nucleotide between those two genes.

### Base composition and codon usage

The base composition bias of an individual strand can be described by skewness [51], where CG-skew = (C - G)/(C + G) and AT-skew = (A - T)/(A + T). The strand encoding most of the proteins (including *cox1*) from the F and M genomes of all unionoid species has strong negative CG- and AT-skews (Table 2). Skews calculated for the opposite strand in all six genomes indicate complementary strand bias, with positive CG- and AT-skew values (Table 2), an expected result since, for example, A-skew on one strand is usually balanced by T-skew on the other [52].

### Transfer RNA genes

In all eight unionoid mt genomes, we identified all 22 tRNA genes according to their secondary structure features and their corresponding anticodons. Most have the potential to fold into a normal cloverleaf structure, although some do not have paired DHU arms, and a few others have mismatched bp. The putative cloverleaf secondary structures of unionoid tRNAs are available in the additional files (Additional file 1, Figures S1, S2, S3, S4, S5, S6, S7 and S8). The tRNA genes are ~60-70 bp long and the mean GC content varies between 35.2% and 37.4%. In the eight mt genomes, most of the tRNA genes are located on the light strand; only *trnH *(Histidine) and *trnD *(Aspartate) are located on the heavy strand along with most of the protein-coding genes. As specified earlier, *trnH *has distinct localizations in F and M unionoid genomes (Figure 3). For all mt genomes (except M *I. japanensis*), the DHU arm of *trnS1 *(Serine) is unpaired. Unpaired DHU arms are also observed for the second Serine *trnS2 *(tct) and Threonine *trnT *in the M genome of *P. grandis*, for the Arginine *trnR *and Threonine *trnT *in the M genome of *V. ellipsiformis *and for the Cysteine *trnC *and Threonine *trnT *in the M genome of *Q. quadrula*. DHU arm for the Lysine *trnK *in the F genome of *Q. quadrula *is also unpaired. No unpaired DHU arm has been observed in tRNAs of M *I. japanensis*.

### Unassigned regions and putative control regions

Twenty-two or thirty-three unassigned regions were detected in the six genomes, with sizes ranging from 1 to 1196 bp. The three newly sequenced F genomes present a more compact arrangement than the three M genomes (Table 3). We observed the opposite trend in *I. japanensis *where the F genome contains more unassigned sequences (12.9% of the genome) than the M genome (8.9% of the genome). The abundance of unassigned sequences in both F and M genomes of the four unionoid species analyzed here is similar with the results observed in their mytiloid F and M counterparts (~10% of unassigned sequences) [31,34,36]. In comparison, the veneroid clam *V. philippinarum *M and F genomes have a higher proportion of unassigned sequences (i.e., > 15.8% for F and > 21.3% for M) (Okazaki M, Ueshima R, personal communication).

**Table 3:** Unassigned regions of the female- and male-transmitted mitochondrial genomes of *Venustaconcha ellipsiformis, Pyganodon grandis, Inversidens japanensis *and *Quadrula quadrula*.

	***V***. *ellipsiformis* Female	***V***. *ellipsiformis* Male	***P***. *grandis* Female	***P***. *grandis* Male	***I***. *japanensis* Female	***I***. *japanensis* Male	***Q***. *quadrula* Female	***Q***. *quadrula* Male
**Unassigned regions**	27	33	24	27	27	22	29	28
**Total (bp)**	1310	1828	1178	2025	2177	1516	1366	1583
**Proportion of the genome (%)**	8.2	10.6	7.3	11.9	12.9	8.9	8.5	9.3
**Largest NC region (bp)**	308	833	480	1103	1196	698	346	555

### Levels of intra- and interspecies sequence divergences

Table 4 contains Intra- and interspecific comparisons of the concatenated nucleotide and amino acid sequences of 12 protein-coding genes (*atp8 *has been excluded and only species for which both F and M genomes are available were used) from the M- and F-transmitted mitochondrial genomes of unionoid bivalves. The smallest nucleotide and amino acid distances are observed between the two F genomes of the most closely-related species, i.e. *V. ellipsiformis *and *Q. quadrula *(P of 0.207 and P(aa) of 0.157). Overall, nucleotide and amino acid sequence divergences between all pairs of F genomes are considerably lower than between M pairs or between the M and F genomes of the same species. Early estimations of the nucleotide divergence between M and F genomes of unionoid bivalves were based on p-distances of partial *cox1 *sequences [20] and were around 28 to 33%. Our results show that these early estimations were conservative and the average uncorrected divergence between the F and M concatenated nucleotide sequences for the 12 mitochondrial protein-coding genes is 41% for *V. ellipsiformis*, 42% for *Q. quadrula *and 43% for *P. grandis *and *I. japanensis*. These very high intraspecific divergences are observed at both the DNA and protein levels (Table 4).

**Table 4:** Intra- and interspecies comparisons for 12 mitochondrial protein-coding genes: average pairwise sequence divergence

Pairs	P	K2P	JC	TrN	Na	P(aa)	D	K_a_	K_s_	**K_a_**/**K_s_**
F × F										
VenF × PygF	0.248	0.306	0.301	0.311	434	0.192	0.214	0.137	1.400	0.098
VenF × InvF	0.249	0.307	0.302	0.312	431	0.191	0.212	0.137	1.358	0.101
VenF × QuaF	0.207	0.246	0.242	0.251	353	0.157	0.170	0.101	1.010	0.100
PygF × InvF	0.274	0.346	0.340	0.353	488	0.216	0.244	0.157	1.785	0.088
PygF × QuaF	0.252	0.312	0.317	0.318	466	0.207	0.231	0.147	1.350	0.109
InvF × QuaF	0.247	0.303	0.308	0.308	466	0.207	0.231	0.144	1.213	0.119
M × M										
VenM × PygM	0.370	0.514	0.510	0.529	950	0.421	0.496	0.353	1.548	0.228
VenM × InvM	0.340	0.459	0.454	0.468	865	0.384	0.484	0.300	1.352	0.222
VenM × QuaM	0.303	0.393	0.389	0.402	796	0.353	0.435	0.272	0.946	0.288
PygM × InvM	0.372	0.522	0.514	0.533	882	0.391	0.496	0.327	2.093	0.156
PygM × QuaM	0.374	0.522	0.518	0.536	933	0.414	0.534	0.360	1.523	0.236
InvM × QuaM	0.340	0.458	0.454	0.465	836	0.371	0.463	0.307	1.241	0.247
F × M										
VenF × VenM	0.407	0.586	0.586	0.603	1135	0.503	0.700	0.474	1.112	0.426
PygF × PygM	0.428	0.636	0.634	0.657	1142	0.506	0.706	0.494	1.428	0.346
InvF × InvM	0.430	0.641	0.638	0.653	1139	0.505	0.703	0.482	1.523	0.316
QuaF × QuaM	0.419	0.625	0.614	0.632	1164	0.516	0.726	0.500	1.142	0.438

NOTE. --P, uncorrected nucleotide divergence; K2P, nucleotide divergence with Kimura's -parametertwo model; JC, nucleotide divergence with Jukes and Cantor model; TrN, nucleotide divergence with Tamura-Nei model; Na, total number of amino acid differences; P(aa), uncorrected amino acid p-distances; D, Poisson-corrected amino acid distances, and estimates of K_a _(the number of nonsynonymous substitutions per nonsynonymous site) and K_s_(t he number of synonymous substitutions per synonymous site) using the Jukes and Cantor (1969) correction. VenF: *Venustaconcha ellipsiformis *F genome; PygF: *Pyganodon grandis *F genome; InvF: *Inversidens japanensis *F genome; QuaF: *Quadrula quadrula *F genome; VenM: *V. ellipsiformis *M genome; PygM: *P. grandis *M genome; InvM: *I. japanensis *M genome; QuaM: *Q. quadrula *M genome.

## Discussion

### Phylogenetic analysis

The seemingly anomalous difference in branching pattern between the M and F *Mytilus *genomes is due to an asymmetric introgression of *M. edulis *M mtDNA into the Baltic *M. trossulus *[36]. Nevertheless, the ingroup (= bivalves) topology in Figure 1 is consistent with other sequence-based phylogenetic reconstructions in that the Unionoida is basal to Pteriomorphia+Veneroida (e.g., [41,45,48]) thus reinforcing the hypothesis that the Unionoida is a relatively ancient bivalve lineage potentially harboring the ancestral characteristics of DUI.

Although the phylogenetic hypothesis ((Pteriomorphia, Veneroida) Unionoida) is not typically portrayed as the best estimate of evolutionary relationships for these lineages at this time (e.g., [53]: Figure six point height), the statistical robustness of our phylogenetic analyses with regards to the Bivalvia (Figure 1) indicates that it should be seriously evaluated in future, higher level bivalve phylogenetic studies.

The three origins of DUI for the taxa included in this study (Figure 2A) runs counter to the prevailing hypothesis of a single origin for this complex trait with subsequent reversals to SMI [23,26-28] but it is not unexpected given the bias toward "DUI absence" stemming from the difficulties in confirming the presence of DUI (e.g., [20,25,26,54]). The complexity of the cyto-nuclear interactions involved in DUI and its very narrow taxonomic distribution are consistent with the hypothesis that the gain of DUI is a relatively rare event with subsequent losses being potentially more common. If a low ratio of rate of DUI gain to rate of DUI loss actually holds, then the use of the Dollo parsimony model [55,56] is more appropriate than the use of the MK1 model and the former indicates a single gain of DUI with three subsequent losses (Figure 2B). A much more accurate understanding of the actual taxonomic distribution of DUI combined with a taxonomically expanded version of our robustly supported bivalve phylogeny (Figure 1) would allow a rigorous evaluation of the single vs. multiple origins of DUI hypotheses as well as the rates of DUI gain vs. loss.

### Genome structural features

Overall, the most notable differences observed between M and F unionoid genomes are (i) the position of *trnH*, (ii) an inversion of the *trnD *and *atp8 *genes, (iii) the length of the *cox2 *gene (the M genomes possess a 3' extension of *cox2*) as well as (iv) a noncoding region between *nd4 *and *nd4l *in the M mtDNA genome of *V. ellipsiformis *(Figure 3). The female-transmitted mtDNAs of *V. ellipsiformis, P. grandis *and *Q. quadrula *are comparable in many respects to the F mtDNA of *L. ornata*, which is unique in gene arrangement relative to all other molluscan and metazoan mt genomes [37]. The mitochondrial gene order rearrangement in the F genomes of *I. japanensis *and *H. cumingii *(Unionoida: Unionidae: Ambleminae: Gonideini), i.e. the relative positions of the *nd2, trnM *to *nd3 *genes, appear to be unique to the Gonideini as neither *L. ornata, V. ellipsiformis *(Unionidae: Unionidae: Ambleminae: Lampsilini), *Q. quadrula *(Unionoida: Unionidae: Ambleminae: Quadrulini) nor *P. grandis *(Unionidae: Unionidae: Unioninae: Anodontini) show this rearrangement. We suggest that this distinct gene order in the F genomes of *I. japanensis *and *H. cumingii *resulted from a tandem duplication of the gene region followed by the deletion of segments of the duplicated gene region. Losses and gains of genes, gene rearrangements and unusually large amounts of duplicated or noncoding nucleotides are common in mollusk mitochondrial genomes [39,57,58].

**Figure 4:**
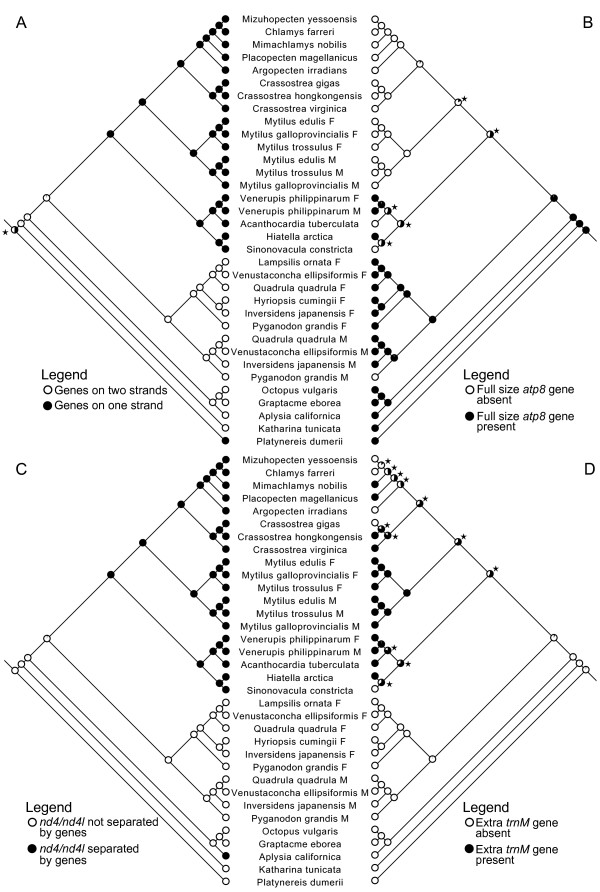
**ML-based ancestral character state reconstructions of mt genomic characteristics on the tree presented in Figure 1**: (A) genes on one strand vs. genes on two strands, (B) presence/absence of full size *atp8 *gene, (C)*nd4/nd4l *separated by genes vs. *nd4/nd4l *not separated by genes, (D) presence/absence of an extra *trnM *gene. For each of these four mt genomic characteristics, the indicated character state for the unionoid bivalve ancestor is identical to that indicated for the molluscan ancestor. "Stars" indicate nodes for which the character state designations were not statistically significant (all other nodes were statistically significant).

When looking across other complete bivalve genomes, which include species from the orders Pectinoida, Ostreoida, Veneroida and Mytiloida (Organellar Genome Retrieval database OGRe; [59]), all genes are characteristically on the same strand (Figure 4A). Among the Bivalvia, only in unionoids are the genes transcribed in both directions. Robust phylogenies are necessary to compare bivalve mt genome arrangements in an evolutionary context and our understanding of the phylogenetic relationships among the major lineages within the Bivalvia and among molluscan classes is still limited and controversial [60]. Nonetheless, based on bivalve phylogenetic analyses presented in Hoeh et al. [45], Giribet and Distel [48], Dreyer and Steiner [41] and herein (Figure 1), the "all-on-one-strand" phenotype likely represents a shared, derived characteristic that evolved once in the common ancestor of the Pteriomorphia and Veneroida with the unionoid model of genes on different strands representing the ancestral state for the Bivalvia (Figure 4A).

#### tRNA Histidine

While the gene boundary *nd3-trH* on the heavy strand observed in unionoid F genomes is not shared by any other mollusk taxon studied so far, the gene boundary *nd5-trnH* observed in the four unionoid M genomes is also shared by nine species of cephalopods, the polyplacophoran *Katharina tunicata *and the gastropod *Haliotis rubra*. This particular gene boundary could represent the ancestral character state for the Mollusca. The tRNA genes are the most evolutionarily mobile elements of the animal mitochondrial genome and variation in mitochondrial tRNA gene organization have been found in multiple divergent taxa [61,62]. Rearrangement of tRNAs occurs frequently because their secondary structure facilitates their translocation [63]; alternatively, rearrangements can also result from a duplication event [64].

#### Extension of the M cytochrome c oxidase subunit II gene

All unionoid bivalve M genomes examined to date contain an M*cox2e *region [25], which is not present in other DUI-possessing bivalve lineages nor, apparently, in any other animal mitochondrial genomes [43]. Structural characterization of the M*COX2e *region predicted the presence of an interspecifically variable number of transmembrane helices [43], and immunohistochemistry- and immunoelectronmicroscopy-based analyses revealed that M*COX2e *is expressed in sperm mitochondria [65] and is sub-cellularly localized to both inner and outer mitochondrial membranes [16]. The latter localization, which possibly "tags" the outer surface of unionoid M genome-bearing mitochondria, could facilitate the differential segregation of the M genome-containing mt, derived from the fertilizing sperm, in male and female embryos (as observed in *Mytilus*; [66,67]). Consistent with the above, seasonal variation in expression profiles suggest that unionoid M*COX2e *functions in reproduction [16,43,64].

#### Atp8 gene

In animal mtDNAs, the *atp8 *gene is the smallest protein-coding gene (≈ 50 to 65 aa) with only a few highly conserved amino acid residues. It encodes a protein subunit of the F_0 _portion of the mitochondrial ATP synthase, which is the enzymatic complex that drives the phosphorylation of ADP to ATP. The ATP synthase comprises the F_1 _catalytic domain situated in the mitochondrial matrix and the F_0 _proton pore embedded in the mitochondrial inner membrane. Although the specific function of *ATP8 *is not yet known, in yeast, it is thought to play an important role in the assembly of the F_0 _portion of ATP synthase and in determining ATP synthase activity (reviewed in [68]). In mammals, it is the most rapidly evolving mitochondrial protein-coding gene [69]. *Atp8 *has been lost independently from the mt genomes of several lineages including some bivalves [e.g., marine mussels possessing DUI [31,34,36] and oysters [70], secernentean nematodes [71], and platyhelminths [72]. Interestingly, all other mollusk species (i.e., gastropods, cephalopods, polyplacophorans and scaphopods) studied to date possess an *atp8 *gene [2,70]. The F and M genomes of the marine clam *Venerupis philippinarum *also possess a short putative *atp8 *gene (37 aa; [41]) and a potential remnant of the *atp8 *gene has been found in the eastern oyster *Crassostrea virginica *[40]. These observations and the phylogeny displayed in Figure 1 reinforce the hypothesis that unionoid mt genomes possess the molluscan ancestral character state (= the presence of *atp8*) and that two losses of *atp8 *in veneroids and another in the common ancestor of the Pteriomorphia could have occurred during bivalve phylogenesis (Figure 4B). In the M genome of *P. grandis*, even though we identified an open reading frame that corresponds to a portion of *atp8*, the complete stop codon early in the sequence could yield a non-functional protein. Further analysis will be necessary to confirm or refute the existence of a functional *atp8 *in the M genome of this species. For now, the presence/absence of *atp8 *seems extremely labile across bivalve taxa, but this phenomenon does not appear to be related to the presence/absence of doubly uniparental inheritance.

#### NADH dehydrogenase subunits 4 and 4L genes

NADH dehydrogenase subunits 4 and 4L genes generally overlap or are adjacent to one another in animal mt genomes [57,73]. This is also the case for most unionoid mt genomes examined in this study except for the M genome of *V. ellipsiformis *where those genes are separated by a large noncoding region as well as for the F genome of *I. japanensis*, where both genes are separated by one nucleotide. We cannot exclude the possibility that this single nucleotide in the F *I. japanensis *sequence represents a sequencing error.

In vertebrates *nd4 *and *nd4l *are transcribed as one bicistronic mRNA, and are therefore localized together [73]. Moreover, in several mollusks (i.e., one scaphopod, some gastropods and all 12 cephalopods studied to date (Organellar Genome Retrieval database OGRe; [59]), these two genes are also adjacent to one another or overlap. However, in all other non-unionoid bivalve species studied to date (7 genera), *nd4 *and *nd4l *have several intervening coding genes (e.g., *Crassostera gigas *[40] and *Hiatella arctica *[41]). Again, the overlap observed between *nd4-nd4l *in most of the new unionoid genomes analyzed herein and the phylogeny displayed in Figure 1 support the hypothesis that unionoid mt genomes possess the molluscan ancestral character state and that the derived state, "intervening genes", occurred once in the common ancestor of pteriomorph and veneroid bivalves (Figure 4C).

### Base composition and codon usage

Although the exact mechanisms responsible for creating CG- and AT-skews like those observed in this study are still poorly understood, it is most likely created by the biases in mutational pressure owing to differences in the time spent as single-stranded DNA during both transcription and replication [74]). The negative CG- and AT-skews observed in the strand that encodes most of the proteins (i.e., *cox1 - cox3 - atp6 - atp8 - nd4L - nd4 - nd5 - nd3 - cox2 *(M*cox2e*)) and that make it G+T rich is reflected in the use of synonymous codons (Additional file 2, Table S1). This is particularly evident at the third codon positions of protein-coding genes where C- and A-ending codons are used less frequently. Overall, TTT (Phe), TTG (Leu) and TTA (Leu) are the most frequent codons, a result consistent with other invertebrate mtDNAs [34]. Except for the stop codons TAA and TAG, TGC, CGC and ACG are among the least used codons. Of these, CGC is also the least common codon in the mtDNA of other mollusks [34].

### Transfer RNA genes

Interestingly, unionoid bivalves do not possess an extra *trnM*, a situation that is present in both pteriomorph and veneroid bivalves [2,31,34]. The presence of an extra *trnM *within the latter two lineages could represent a character state that was (1) independently derived multiple times, (2) derived once with multiple independent secondary losses or (3) both derived and lost multiple times independently (Figure 4D). The absence of a second *trnM *in all other molluscan species studied to date [2] reinforces the hypothesis that unionoids likely possess the molluscan ancestral character state for this character (Figure 4D).

### Unassigned regions and putative control regions

The presence of multiple unassigned regions is not uncommon in mollusk mitochondrial genomes [75,76] and is usually suggestive of molecular rearrangements [77]. The large unassigned region located between *nd5 *and *trnQ *in F genomes and between *trnH *and *trnQ *in M genomes has been identified as a potential heavy strand origin of replication (OH) [78]. Otherwise, F and M unionoid mitochondrial genomes appear to contain multiple and potentially bidirectional OL control regions [78].

### Levels of intra- and interspecies sequence divergences

It should be stressed that the measured divergences between unionoid F and M genomes considerably surpass intra- or inter-species values reported in classical model systems used for the study of intergenomic co-evolution [9,79,80]. From a nucleo-mitochondrial evolutionary perspective, the question of how male freshwater mussels can tolerate heteroplasmy characterized by such variability remains to be solved.

Among species with DUI, freshwater mussels exhibit the greatest nucleotide and amino acid divergences between their gender-associated mtDNAs. For example, the average uncorrected nucleotide divergence observed between the F and M concatenated sequences of the 12 mitochondrial protein-coding genes of the marine mussel *Mytilus edulis *is about 23% [31]. The smaller level of divergence observed between the M and F mtDNAs in *Mytilus *is likely associated with periodic "role-reversal" or "masculinization" events, which are characterized by an invasion of the male route of inheritance by an F-like genome that becomes transmitted through sperm as a standard M genome [14,27,81,82]. Specifically, the F-like, "recently-masculinized" M genome is only significantly different from a standard *Mytilus *F genome in that it possesses a so-called "standard M genome control region" and, as it's name implies, it is paternally transmitted (see [31] and [83] for details). Therefore, such masculinization events reset to zero the level of mitochondrial gene sequence divergence between the M and F genomes. Complete absence of masculinization events, for over 200 million years, can explain the considerably greater divergences between unionoid M and F mtDNAs [22,25,42]. It has been proposed that the unionoid M-specific extension of the cytochrome *c *oxidase subunit II gene represents such specialization of the unionoid M genome that recombination (i.e. the addition of an M type genome's control region to an F genome) leading to role reversals are no longer possible in this taxon [83].

According to our results, ~50% amino acid divergence between unionoid F and M genomes can be tolerated by a species' nuclear environment without any major disruption of cytonuclear co-adaptation or impairment of mitochondrial function. This level of divergence could hardly be explained only by relaxation of selective pressure induced by a loss of metabolic function of M mtDNA since two recent studies have clearly shown the importance of M mtDNA gene products on sperm performance in *Mytilus edulis *[84,85]. Further characterization of the conserved versus radical amino acid changes in evolutionarily conserved or non-conserved positions of mitochondrial proteins will help to delineate the levels/types of divergence in mtDNA encoded peptides that can be tolerated by a species' nuclear genome.

## Conclusions

The basal position of the Unionoida within the autolamellibranch bivalves (Figure 1) and the hypothesized single origin of DUI (Figure 2B; [23,26-28]) suggest that (1) DUI arose in the ancestral autolamellibranch bivalve lineage and was subsequently lost in multiple descendant lineages and (2) the DUI characteristics observed in unionoid bivalves could more closely resemble the DUI ancestral condition. We described the general features of eight mt genomes from unionoid bivalve species with the doubly uniparental mode of mitochondrial inheritance and highlighted several unusual characteristics of the M genomes, compared to their female-transmitted counterparts, e.g., the presence of M*cox2e *and a novel localization of *trnH*. Based on the concatenated nucleotide sequences of 12 mitochondrial protein-coding genes, we determined an uncorrected amino acid p-distance between the M and F genomes of ~50%. From a nucleo-mitochondrial functional perspective, the question of how male freshwater mussels can tolerate heteroplasmy characterized by such variability remains to be solved as does the function(s) of DUI. Finally, the presence of the M*cox2e *is one important feature that distinguishes markedly, but not solely, the unionoid M from the F genomes, but also the unionoid M from all other DUI-possessing bivalves as well as all other metazoan mtDNAs. This suggests that it could have been a facilitator of the transition from SMI to DUI in the ancestral autolamellibranch (assuming a single origin of DUI) or ancestral unionoid (assuming multiple origins of DUI) lineage. If the former hypothesis is corroborated, M*cox2e *was subsequently lost from the M genome in the ancestor of the Pteriomorphia+Veneroida. Irrespective of a single vs. multiple origins of DUI, the ancestral character state reconstructions in Figure 4 imply that significant mt genomic reorganization occurred in the Bivalvia subsequent to the divergence of the unionoid lineage. Studying additional complete bivalve mt genomes will give us the best hope of unraveling the origin(s) and function(s) of DUI as well as the origins and consequences of the unique mt genomic variation in the Bivalvia.

### DNA extraction, PCR amplification and sequencing

Mitochondrial DNA was extracted from one male and one female *Venustaconcha ellipsiformis *from Gladwin County, Michigan (USA), one male and one female *Pyganodon grandis *from the northern Radisson region of Québec (Canada) (48°18'24"N, 68°29'34"W) and one male and one female *Quadrula quadrula *from the White River, Indiana (USA). Microscopic examination of gonadal tissues was performed to determine mussel gender based on the presence of eggs or sperm/sperm morulae. Total genomic DNA extractions were performed on the tissues (gonadal tissue for the male and the female *P. grandis *and gonadal tissue for the males and mantle tissue for the females *V. ellipsiformis *and *Q. quadrula*) using a QIAGEN DNA Extraction Kit (QIAGEN Inc., Mississauga, Canada) following the manufacturer's protocol. Subsequently, each mitochondrial genome was amplified by long-PCR from the genomic DNA in two large overlapping regions using the F-specific primer FCOIIhFor (5'-GCCTTATGGGGTTGATAGGCGAGTTCTTGTGAGG-3') with the amblemine-specific primer Ambl16SFor (5'-CTGGGTTTGCGACCTCGATGTTGGCTTAGGGAAA-3'), and the F-specific primer FCOI195R (5'-GCATAACAATTTCACACAGGCCAATCATYATWGGYATNACCA-3') with the amblemine-specific Ambl16SRev (5'-TTTCCCTAAGCCAACATCGAGGTCGCAAACCCAG-3') for the F genomes of *V. ellipsiformis *and *Q. quadrula*, and the M-specific primer MCOIIh63F (5'-CACGACGTTGTAAAACGACTTTATRCCTRTKTGTGTRGARGCTGT-3') with Ambl16SFor, and the M-specific MCOI19R (5'-GGATAACAATTTCACGGGTCCCAATATCYTTATGRTTAGT-3') with Ambl16SRev for the M genomes of *V. ellipsiformis *and *Q. quadrula*. Both the F and M genomes of these species resulted in two fragments of ~11 kb and ~5.5 kb, respectively. The F genome of *P. grandis *was also amplified in two large overlapping regions using the F-specific primer 5'FCOIPygR (5'-TGCCARTAACAARTAYAAAGTA-3') with ND1R (5-GCTATTAGTAGGTCGTATCG-3; [39,86]) and the F-specific primer UNIOND3 (5'-AGHSCKTTTGARTGYGGKTTTGA-3') with ND1F (5-TGGCAGAAAAGTGCATCAGATTTAAGC-3; [39,87]). We obtained two fragments of ~11 kb and ~7 kb, respectively. The M genome of *P. grandis *was amplified with PygMcox2eF (5'-TTGAAGCAGTTAGAGTTGAGGT-3') in combination with 16Sar-L2 (5'-CGCCTGTTTAYCAAAAACAT-3'; modified from [88]) and PygMcox2eR (5'-TAYAATCTTYCAATRTCYTTATGATT-3') combined with 16Sbr-H2 (5'-CCGGTCTGAACTCAGATCRYGT-3'; modified from [88]). PygMcox2eF and PygMcox2eR were specifically designed to amplify the M genome. The resulting fragments were about ~12 kb and ~5.5 kb.

Long-PCR amplifications were performed in 50 μl reaction volumes using the QIAGEN LongRange PCR Kit in similar conditions to the manufacturers' suggestions: 1× LongRange PCR Buffer with 2.5 mM of Mg2+, 500 μM of each dNTP, 0.4 μM each primer, 2 U of LongRange PCR Enzyme Mix and ~25 ng of template DNA. For the M and the F genomes of *V. ellipsiformis *and *Q. quadrula*, reactions were cycled at 85°C for 60 s, 93°C for 60 s, and 37 cycles of 93°C for 15 s, 53°C for 30 s and 68°C for 6 min for the short fragment or 11 min for the longest one. Thermal cycling conditions for the M and F genomes of *P. grandis *were as follows: 93°C for 3 min, followed by 35 cycles of 93°C for 15 s, 46-54°C for 30 s and 68°C for 7-12 min and a final extension at 72°C for 10 min. Each amplicon appeared as one abundant band of the appropriate size on an agarose gel. The resulting PCR products were gel purified using QIAGEN QIAquick Gel Extraction Kit. Following DNA quantification for each amplicon, the two amplicons (~5 μg from each) for each genome were pooled and then processed for direct sequencing in a single reaction by the 454 Life Sciences Massively Parallel Pyrosequencing Platform (whole genome sequencing protocol) of the McGill University and Genome Quebec Innovation Center.

For the M and the F genomes of *V. ellipsiformis*, amplifications were pooled and a total of 10,413 reads were produced to provide at least 45× coverage of the complete mitochondrial genomes. The sequences were then assembled into a single contig of 15,975 base pairs (bp) for the F genome and 17,174 bp for the M genome. For the M and the F genomes of *Q. quadrula*, amplifications were pooled and a total of 11,978 reads were produced to provide at least 66× coverage of the complete mitochondrial genomes. The sequences were assembled into a single contig of 16,033 bp for the F genome and 16,970 bp for the M genome. For the *P. grandis *F genome, draft assemblies were based on 14,794 total reads. The initial assembly of the 454 pyrosequencing data into two predominant contigs (~6.7 kb) and a small one (834 bp) was provided by *454 Life Sciences *(Branford, CT, USA), and corresponded to a mitochondrial genome coverage of 115× and 437× respectively. The final assembly in one large contig of 15,848 bp was performed using SeqMan (DNAStar Inc., Madison, WI, USA). The complete M genome of *P. grandis *was generated from assembly of 7,652 successful sequence reads into a single contig of 17,071 bp which corresponded to an overall mitochondrial genome coverage of > 100×.

The complete sequences of the F and M mitochondrial genomes for *Venustaconcha ellipsiformis, Pyganodon grandis *and *Quadrula quadrula *can be accessed under the GenBank accession numbers FJ809753, FJ809752, FJ809754, FJ809755, FJ809750, and FJ809751, respectively.

### Gene annotation and analysis

The complete F and M mitochondrial genomes for each species were initially analyzed with the NCBI Open Reading Frame Finder using the invertebrate mitochondrial code. Protein-coding and ribosomal RNA genes were annotated using DOGMA [88] and then aligned with the mtDNA genes annotated in GenBank using ClustalW [89]. The 5' and 3' ends of both *rrnL *and *rrnS *genes were assumed to be adjacent to the ends of bordering tRNA genes. Mitochondrial tRNA genes were identified and confirmed using a combination of programs: tRNAscan-SE 1.21 [90] with a COVE cutoff score of 0.1, DOGMA [89] and ARWEN [91]. Mitochondrial gene order comparisons were facilitated by the use of the OGRe web site at http://drake.physics.mcmaster.ca/ogre/index.shtml[59].

Basic sequence statistics and evolutionary distances among genes were performed using MEGA version 4.0 [92] and DnaSP version 4.0 [93]. To estimate evolutionary distance between pairwise comparisons, the following parameters were used: uncorrected nucleotide divergence (Pi = uncorrected nucleotide diversity), nucleotide divergence using the Jukes-Cantor (JC), Kimura two-parameter (K2P), and Tamura and Nei (TrN) models of nucleotide substitution. Estimated parameters also included total amino acid differences (Na), uncorrected amino acid distances (p(aa)), poisson-corrected amino acid distances (D), number of synonymous substitutions per synonymous site (K_s_) and number of nonsynonymous substitution per nonsynonymous site (K_a_) [94]. The Jukes-Cantor correction for multiple substitutions was applied. Strand asymmetry was measured using the formulas AT-skew = (A - T)/(A + T) and CG-skew = (C - G)/(C + G) [51,95] and calculated with MEGA 4.0 [92] at fourfold redundant sites for each mitochondrial protein-coding gene.

Phylogenetic trees for the Bivalvia, using Bayesian inference (BI), maximum likelihood (ML) and maximum parsimony (MP), were constructed using concatenated nucleotide and amino acid sequences from 12 protein-coding genes (we excluded *atp8 *due to alignment issues and its apparent absence in multiple bivalve species). We used both Clustal W [96] and Dialign version 2.2.1 [97] for the alignments, with subsequent manual adjustments, and the amino acid alignment was used as a template to align the corresponding codons. Amino acids from 29 complete bivalve mitochondrial genomes and those from five outgroup species (gastropod *Aplysia californica *[NC_005827], cephalopod *Octopus vulgaris *[NC_006353], scaphopod *Graptacme eborea *[NC_006162], polyplacophoran, *Katharina tunicata *[NC_001636], polychaete *Platynereis dumerii *[NC_000931]) were aligned using Clustal W in MEGA 4.0 and manually reviewed (Table 1). Regions of ambiguous alignment were excluded prior to the phylogenetic analyses. The analyzed matrices had either 7,704 nucleotide positions or 2,568 amino acid positions and these files are available from the authors.

The codon- and amino acid-based BI analyses were conducted with Mr. Bayes (v. 3.1.2; [98,99]). The codon-based analysis invoked the M3 model [100] with two simultaneous runs of 5 million generations each (a total of 50,000 saved trees/run). The amino acid-based BI analysis invoked the variable rate "glorified GTR model" (see the MrBayes manual;[101]) with two simultaneous runs of 2.9 million generations each (a total of 29,000 saved trees/run). Both sets of BI analyses reached convergence (average standard deviation of the split frequencies was <0.01) and the burnin for each set was determined by reference to the log probability of observing the data × generation plot (codon-based BI run burnin = 4 million generations [= the last 10,000 trees/run saved contributed to the majority-rule tree], amino acid-based BI run burnin = 1.9 million generations [= the last 10,000 trees/run saved contributed to the majority-rule tree]).

Codon and amino acid-based ML analyses were conducted with Garli (v. 0.96;[102]). The M3 model was used in the codon-based ML analysis which was set to use the observed nucleotide frequencies at each codon position separately. A non-parametric bootstrap [103] analysis was performed, using 300 replicates, to assess nodal support for the codon analysis-based trees. The program ProtTest http://darwin.uvigo.es/software/prottest.html was used to evaluate the best amino acid model for our data from those models available in Garli. Both the Akaike information criterion and Bayesian information criterion selected the WAG+F model [104] as the best available model which was therefore used in the ML amino acid analysis. A non-parametric bootstrap was performed, using 600 replicates, to assess nodal support for the a.a. analysis-based trees.

Maximum parsimony analyses were conducted with PAUP* [105]. The nucleotide-based MP analysis utilized equally weighted transversion parsimony (= only purines vs. pyrimidines were coded) and 1000 random addition runs for estimating the most parsimonious tree. A non-parametric bootstrap transversion parsimony analysis was run (with 1000 replicates) using 10 random addition runs per replicate. The amino acid-based MP analysis was carried out with equal weighting and 1000 random addition runs were used to estimate the most parsimonious tree. Lastly, an equally weighted parsimony, non-parametric, bootstrap analysis was run on the a.a. matrix (with 1000 replicates) using 10 random addition runs per replicate.

The estimation of ancestral mitogenomic character states and the presence/absence of DUI, based on the majority-rule codon-based BI tree, was carried out using the ML algorithm in Mesquite (v.2.6; [106]). An estimation of ancestral character states for the presence/absence of DUI, using the best codon-based BI tree and Dollo parsimony, was done with MacClade (v.4.07;[107]). The asymmetry likelihood ratio test was used to determine whether the AsymmMK model was significantly better than the MK1 model (see the Mesquite manual). The MK1 model was used in all likelihood reconstructions because in all cases, the AsymmMK model was not a significantly better model, therefore we used the simpler model (the MK1 model has one less parameter). The use of a likelihood ratio test to calculate *P*-values for ancestral states is not possible because hypotheses regarding the likelihoods of each possible state at a given node are non-nested. Therefore, to make decisions regarding the significance of ancestral character states Pagel ([108] following [109]), recommended that ancestral character state estimates with a log likelihood two or more units lower than the best state estimate (decision threshold [T] set to T = 2) be rejected. Generally viewed as a conservative cutoff, this threshold has been used by numerous recent authors (e.g., [110-112]). For the data presented herein, this protocol ensures that all of the character states judged to be significant have proportional likelihoods at least 10 times greater than that of any other state.

## Authors' contributions

HDB and SB conceived the study, sequenced and annotated the new mitochondrial genomes. HDB aligned the sequence, analyzed the data, and drafted the manuscript. EGC conducted the computational phylogenetic analysis. PUB participated in coordination of the study and also helped to improve the manuscript. AEB provided tissue samples for DNA extraction and material support for the sequencing. DTS helped in interpreting the data and revising the manuscript. WRH also conceived the study and participated in its design, in data analysis and in drafting the manuscript. All authors read and approved the final manuscript.

## Supplementary Material

Additional file 1 **Supplemental figures**. **Figure S1**. Inferred secondary structures of the 22 mitochondrial tRNAs from F *Venustaconcha ellipsiformis*, shown in the order they occur in the genome, beginning with *trnH*. Amino acid identities are given above each sequence. **Figure S2**. Inferred secondary structures of the 22 mitochondrial tRNAs from M *Venustaconcha ellipsiformis*, shown in the order they occur in the genome, beginning with *trnA*. Amino acid identities are given above each sequence. **Figure S3**. Inferred secondary structures of the 22 mitochondrial tRNAs from F *Pyganodon grandis*, shown in the order they occur in the genome, beginning with *trnH*. Amino acid identities are given above each sequence. **Figure S4**. Inferred secondary structures of the 22 mitochondrial tRNAs from M *Pyganodon grandis*, shown in the order they occur in the genome, beginning with *trnA*. Amino acid identities are given above each sequence. **Figure S5**. Inferred secondary structures of the 22 mitochondrial tRNAs from F *Inversidens japanensis*, shown in the order they occur in the genome, beginning with *trnH*. Amino acid identities are given above each sequence. **Figure S6**. Inferred secondary structures of the 22 mitochondrial tRNAs from M *Inversidens japanensis*, shown in the order they occur in the genome, beginning with *trnA*. Amino acid identities are given above each sequence. **Figure S7**. Inferred secondary structures of the 22 mitochondrial tRNAs from F *Quadrula quadrula*, shown in the order they occur in the genome, beginning with *trnH*. Amino acid identities are given above each sequence. **Figure S8**. Inferred secondary structures of the 22 mitochondrial tRNAs from M *Quadrula quadrula*, shown in the order they occur in the genome, beginning with *trnA*. Amino acid identities are given above each sequence. Click here for file

Additional file 2 **Table S1**. Codon usage in the female- and male-transmitted mitochondrial genomes of *Venustaconcha ellipsiformis, Pyganodon grandis, Inversidens japanensis *and *Quadrula quadrula*. Table of the codon usage in the female- and male-transmitted mitochondrial genomes of *Venustaconcha ellipsiformis, Pyganodon grandis, Inversidens japanensis *and *Quadrula quadrula*. Click here for file

## References

[B1] Boore JL Animal mitochondrial genomes Nucl Acids Res 1999 27 1767 1780 10.1093/nar/27.8.1767 10101183 PMC148383

[B2] Attardi G Animal mitochondrial DNA: an extreme example of genetic economy Int Rev Cytol 1985 93 93 145 full_text 3891661 10.1016/s0074-7696(08)61373-x

[B3] Gissi C Iannelli F Pesole G Evolution of the mitochondrial genome of Metazoa as exemplified by comparison of congeneric species Heredity 2008 101 301 320 10.1038/hdy.2008.62 18612321

[B4] Xu JP The inheritance of organelle genes and genomes: patterns and mechanisms Genome 2005 48 951 958 10.1139/g05-082 16391664

[B5] Elson JL Lightowlers RN Mitochondrial DNA clonality in the dock: can surveillance swing the case? Trends Genet 2006 22 603 607 10.1016/j.tig.2006.09.004 16979783

[B6] Birky CW The inheritance of genes in mitochondria and chloroplasts: Laws, mechanisms, and models Annu Rev Genet 2001 35 125 148 10.1146/annurev.genet.35.102401.090231 11700280

[B7] Rand DM The units of selection on mitochondrial DNA Annu Rev Ecol Syst 2001 32 415 448 10.1146/annurev.ecolsys.32.081501.114109

[B8] Shoubridge EA Wai T MEDICINE: Sidestepping Mutational Meltdown Science 2008 319 914 915 10.1126/science.1154515 18276880

[B9] Breton S Doucet-Beaupré H Stewart DT Hoeh WR Blier PU The unusual system of doubly uniparental inheritance of mtDNA: isn't one enough? Trends Genet 2007 23 465 474 10.1016/j.tig.2007.05.011 17681397

[B10] Passamonti M Ghiselli F Doubly uniparental inheritance: two mitochondrial genomes, one precious model for organelle DNA inheritance and evolution DNA Cell Biol 2009 28 79 89 10.1089/dna.2008.0807 19196051

[B11] Sutherland B Stewart D Kenchington ER Zouros E The fate of paternal mitochondrial DNA in developing female mussels, *Mytilus edulis *: Implications for the mechanism of doubly uniparental inheritance of mitochondrial DNA Genetics 1998 148 341 347 9475744 10.1093/genetics/148.1.341 PMC1459795

[B12] Obata M Sano N Kawamura K Komaru A Inheritance of two M type mitochondrial DNA from sperm and unfertilized eggs to offspring in *Mytilus galloprovincialis* Dev Growth Differ 2007 49 335 344 17501909 10.1111/j.1440-169X.2007.00930.x

[B13] Garrido-Ramos MA Stewart DT Sutherland BW Zouros E The distribution of male-transmitted and female-transmitted mitochondrial DNA types in somatic tissues of blue mussels: Implications for the operation of doubly uniparental inheritance of mitochondrial DNA Genome 1998 41 818 824 10.1139/gen-41-6-818

[B14] Venetis C Theologidis I Zouros E Rodakis GC No evidence for presence of maternal mitochondrial DNA in the sperm of *Mytilus galloprovincialis *males Proc R Soc London Ser B 2006 273 2483 2489 10.1098/rspb.2006.3607 PMC1634914 16959639

[B15] Obata M Kamiya C Kawamura K Komaru A Sperm mitochondrial DNA transmission to both male and female offspring in the blue mussel *Mytilus galloprovincialis* Dev Growth Differ 2006 48 253 261 10.1111/j.1440-169X.2006.00863.x 16681650

[B16] Chakrabarti R Walker JM Chapman EG Shepardson SP Trdan RJ Curole JP Watters Stewart DT Vijayaraghavan S Hoeh WR Reproductive function for a C-terminus extended, male-transmitted cytochrome c oxidase subunit II protein expressed in both spermatozoa and eggs FEBS Lett 2007 581 5213 5219 10.1016/j.febslet.2007.10.006 17950289 PMC2141648

[B17] Rawson PD Hilbish TJ Evolutionary relationships among the male and female mitochondrial-DNA lineages in the Mytilus edulis species complex Mol Biol Evol 1995 12 893 901 7476135 10.1093/oxfordjournals.molbev.a040266

[B18] Stewart DT Saavedra C Stanwood RR Ball AO Zouros E Male and female mitochondrial DNA lineages in the blue mussel *Mytilus* Mol Biol Evol 1995 12 735 747 7476121 10.1093/oxfordjournals.molbev.a040252

[B19] Stewart DT Kenchington ER Singh RK Zouros E Degree of selective constraint as an explanation of the different rates of evolution of gender-specific mitochondrial DNA lineages in the mussel *Mytilus* Genetics 1996 143 1349 1357 8807306 10.1093/genetics/143.3.1349 PMC1207403

[B20] Hoeh WR Stewart DT Sutherland BW Zouros E Multiple origins of gender-associated mitochondrial DNA lineages in bivalves (Mollusca: Bivalvia) Evolution 1996 50 2276 2286 10.2307/2410697 28565656

[B21] Passamonti M Scali V Gender-associated mitochondrial DNA heteroplasmy in the venerid clam *Tapes philippinarum *(Mollusca Bivalvia) Curr Genet 2001 39 117 124 10.1007/s002940100188 11405096

[B22] Curole JP Kocher TD Ancient sex-specific extension of the cytochrome c oxidase II gene in bivalves and the fidelity of doubly-uniparental inheritance Mol Biol Evol 2002 19 1323 1328 12140244 10.1093/oxfordjournals.molbev.a004193

[B23] Hoeh WR Stewart DT Guttman SI High fidelity of mitochondrial genome transmission under the doubly uniparental mode of inheritance in freshwater mussels (Bivalvia: Unionoidea) Evolution 2002 56 2252 2261 12487355 10.1111/j.0014-3820.2002.tb00149.x

[B24] Passamonti M Boore JL Scali V Molecular evolution and recombination in gender-associated mitochondrial DNAs of the manila clam *Tapes philippinarum* Genetics 2003 164 603 611 12807780 10.1093/genetics/164.2.603 PMC1462575

[B25] Walker JM Curole JP Wade DE Chapman EG Bogan AE Watters GT Hoeh WR Taxonomic distribution and phylogenetic utility of gender-associated mitochondrial genomes in the Unionoida (Bivalvia) Malacologia 2006 48 265 282

[B26] Theologidis I Fodelianakis S Gaspar MB Zouros E Doubly uniparental inheritance (DUI) of mitochondrial dna in *Donax trunculus *(Bivalvia: Donacidae) and the problem of its sporadic detection in Bivalvia Evolution 2008 62 959 970 10.1111/j.1558-5646.2008.00329.x 18208565

[B27] Zouros E The exceptional mitochondrial DNA system of the mussel family Mytilidae Genes Genet Syst 2000 75 313 318 10.1266/ggs.75.313 11280005

[B28] Obata M Shimizu M Sano N Komaru A Maternal inheritance of mitochondrial DNA (mtDNA) in the Pacific oyster (*Crassostrea gigas*): a preliminary study using mtDNA sequence analysis with evidence of random distribution of MitoTracker-stained sperm mitochondria in fertilized eggs Zool Sci 2008 25 248 254 10.2108/zsj.25.248 18393561

[B29] Hoeh WR Stewart DT Sutherland BW Zouros E Cytochrome c oxidase sequence comparisons suggest an unusually high rate of mitochondrial DNA evolution in *Mytilus *(Mollusca: Bivalvia) Mol Biol Evol 1996 13 418 421 8587506 10.1093/oxfordjournals.molbev.a025600

[B30] Liu HP Mitton JB Wu SK Paternal mitochondrial DNA differentiation far exceeds maternal mitochondrial DNA and allozyme differentiation in the freshwater mussel, *Anodonta grandis grandis* Evolution 1996 50 952 957 10.2307/2410870 28568930

[B31] Breton S Burger G Stewart DT Blier PU Comparative analysis of gender-associated complete mitochondrial genomes in marine mussels (*Mytilus *spp.) Genetics 2006 172 1107 1119 10.1534/genetics.105.047159 16322521 PMC1456209

[B32] Hoffman RJ Boore JL Brown WM A novel mitochondrial genome organization for the blue mussel, *Mytilus edulis* Genetics 1992 131 397 412 1386586 10.1093/genetics/131.2.397 PMC1205014

[B33] Boore JL Medina M Rosenberg LA Complete sequences of the highly rearranged molluscan mitochondrial genomes of the scaphopod *Graptacme eborea *and the bivalve *Mytilus edulis* Mol Biol Evol 2004 21 1492 1503 10.1093/molbev/msh090 15014161

[B34] Mizi A Zouros E Moschonas N Rodakis GC The complete maternal and paternal mitochondrial genomes of the mediterranean mussel *Mytilus galloprovincialis *: Implications for the doubly uniparental inheritance mode of mtDNA Mol Biol Evol 2005 22 952 967 10.1093/molbev/msi079 15647523

[B35] Venetis C Theologidis I Zouros E Rodakis GC A mitochondrial genome with a reversed transmission route in the Mediterranean mussel *Mytilus galloprovincialis* Gene 2007 406 79 90 17611047 10.1016/j.gene.2007.06.001

[B36] Zbawicka M Burzynski A Wenne R Complete sequences of mitochondrial genomes from the Baltic mussel *Mytilus trossulus* Gene 2007 406 191 198 17980515 10.1016/j.gene.2007.10.003

[B37] Serb JM Lydeard C Complete mtDNA sequence of the north American freshwater mussel, *Lampsilis ornata *(Unionidae): An examination of the evolution and phylogenetic utility of mitochondrial genome organization in bivalvia (Mollusca) Mol Biol Evol 2003 20 1854 1866 10.1093/molbev/msg218 12949150

[B38] Valles Y Boore JL Lophotrochozoan mitochondrial genomes Integr Comp Biol 2006 46 544 557 10.1093/icb/icj056 21672765

[B39] Smith DR Snyder M Complete mitochondrial DNA sequence of the scallop *Placopecten magellanicus *: Evidence of transposition leading to an uncharacteristically large mitochondrial genome J Mol Evol 2007 65 380 391 10.1007/s00239-007-9016-x 17922075

[B40] Milbury CA Gaffney PM Complete mitochondrial DNA sequence of the eastern oyster *Crassostrea virginica* Mar Biotechnol 2005 7 697 712 10.1007/s10126-005-0004-0 16132463

[B41] Dreyer H Steiner G The complete sequence and gene organization of the mitochondrial genomes of the heterodont bivalves *Acanthocardia tuberculata *and *Hiatella arctica *and the first record for a putative Atpase subunit 8 gene in marine bivalves Front Zool 2006 3 13 10.1186/1742-9994-3-13 16948842 PMC1570459

[B42] Curole JP Kocher TD Evolution of a unique mitotype-specific protein-coding extension of the cytochrome c oxidase II gene in freshwater mussels (Bivalvia: Unionoida) J Mol Evol 2005 61 381 389 10.1007/s00239-004-0192-7 16082567

[B43] Chapman EG Piontkivska H Walker JM Stewart DT Curole JP Hoeh WR Extreme primary and secondary protein structure variability in the chimeric male-transmitted cytochrome c oxidase subunit II protein in freshwater mussels: Evidence for an elevated amino acid substitution rate in the face of domain-specific purifying selection BMC Evol Biol 2008 8 165 10.1186/1471-2148-8-165 18513440 PMC2430956

[B44] Healy JM Spermiogenesis and spermatozoa in the relict bivalve genus *Neotrigonia *: relevance to trigonioid relationships, particularly with Unionoidea Mar Biol 1989 103 75 85 10.1007/BF00391066

[B45] Hoeh WR Black MB Gustafson R Bogan AE Lutz RA Vrijenhoek RC Testing alternative hypotheses of *Neotrigonia *(Bivalvia: Trigonioida) phylogenetic relationships using cytochrome C oxidase subunit I DNA sequences Malacologia 1998 40 267 278

[B46] Graf DL O'Foighil D The evolution of brooding characters among the freshwater pearly mussels (Bivalvia: Unionoidea) of North America J Mollus Stud 2000 66 157 170 10.1093/mollus/66.2.157

[B47] Giribet G Wheeler W On bivalve phylogeny: a high-level analysis of the Bivalvia (Mollusca) based on combined morphology and DNA sequence data Invert Biol 2002 121 271 324

[B48] Giribet G Distel DL Lydeard C, Lindberg DR Bivalve phylogeny and molecular data Molecular Systematics and Phylogeography of Mollusks 2003 Washington DC; Smithsonian Books 45 90

[B49] Graf DL Cummings KS Palaeoheterodont diversity (Mollusca: Trigonioida + Unionoida): what we know and what we wish we knew about freshwater mussel evolution Zool J Linn Soc 2006 148 343 394 10.1111/j.1096-3642.2006.00259.x

[B50] Grande C Templado J Zardoya R Evolution of gastropod mitochondrial genome arrangements BMC Evol Biol 2008 8 61 10.1186/1471-2148-8-61 18302768 PMC2291457

[B51] Perna NT Kocher TD Patterns of nucleotide composition at fourfold degenerate sites of animal mitochondrial genomes J Mol Evol 1995 41 353 358 10.1007/BF01215182 7563121

[B52] Francino MP Ochman H Strand asymmetries in DNA evolution Trends Genet 1997 13 240 245 10.1016/S0168-9525(97)01118-9 9196330

[B53] Giribet G Ponder W, Lindberg DR Bivalvia Phylogeny and Evolution of the Mollusca 2008 Berkeley, CA: University of California Press 105 142

[B54] Krebs RA Combining paternally and maternally inherited mitochondrial DNA for analysis of population structure in mussels Mol Ecol 2004 13 1701 1705 10.1111/j.1365-294X.2004.02133.x 15140112

[B55] Le Quesne WJ The uniquely evolved character concept and its cladistic application Syst Zool 1974 23 513 517 10.2307/2412469

[B56] Farris JS Phylogenetic analysis under Dollo's Law Syst Zool 1977 26 77 88 10.2307/2412867

[B57] Boore JL The complete sequence of the mitochondrial genome of *Nautilus macromphalus *(Mollusca: Cephalopoda) BMC Genomics 2006 7 182 10.1186/1471-2164-7-182 16854241 PMC1544340

[B58] Knudsen B Kohn AB Nahir B McFadden CS Moroz LL Complete DNA sequence of the mitochondrial genome of the sea-slug, *Aplysia californica *: Conservation of the gene order in Euthyneura Mol Phylogenet Evol 2006 38 459 469 10.1016/j.ympev.2005.08.017 16230032

[B59] Jameson D Gibson AP Hudelot C Higgs PG OGRe: a relational database for comparative analysis of mitochondrial genomes Nucl Acids Res 2003 31 202 206 10.1093/nar/gkg077 12519982 PMC165524

[B60] Giribet G Dunn CW Edgecombe GD Kristensen RM Hejnol A Pleijel F Rouse GW Sorensen MV Worsaae K A new dimension in combining data? The use of morphology and phylogenomic data in metazoan systematics J Morphol 2008 269 1462 1462

[B61] Boore JL Lavrov DV Brown WM Gene translocation links insects and crustaceans Nature 2006 392 667 668 10.1038/33577 9565028

[B62] Dowton M Austin AD Evolutionary dynamics of a mitochondrial rearrangement "hot spot" in the Hymenoptera Mol Biol Evol 1999 16 298 309 10028295 10.1093/oxfordjournals.molbev.a026111

[B63] Mueller RL Boore JL Molecular mechanisms of extensive mitochondrial gene rearrangement in plethodontid salamanders Mol Biol Evol 2005 22 2104 2112 10.1093/molbev/msi204 15987876

[B64] Cantatore P Gadaleta MN Roberti M Saccone C Wilson AC Duplication and remoulding of tRNA genes during the evolutionary rearrangement of mitochondrial genomes Nature 1987 329 853 855 10.1038/329853a0 3670390

[B65] Chakrabarti R Walker JM Stewart DT Trdan RJ Vijayaraghavan S Curole JP Hoeh WR Presence of a unique male-specific extension of C-terminus to the cytochrome c oxidase subunit II protein coded by the male-transmitted mitochondrial genome of *Venustaconcha ellipsiformis *(Bivalvia: Unionoidea) FEBS Lett 2006 580 862 866 10.1016/j.febslet.2005.12.104 16414043

[B66] Cao L Kenchington E Zouros E Differential segregation patterns of sperm mitochondria in embryos of the blue mussel *(Mytilus edulis*) Genetics 2004 166 883 894 10.1534/genetics.166.2.883 15020473 PMC1470727

[B67] Cogswell AT Kenchington ELR Zouros E Segregation of sperm mitochondria in two- and four-cell embryos of the blue mussel *Mytilus edulis *: implications for the mechanism of doubly uniparental inheritance of mitochondrial DNA Genome 2006 49 799 807 10.1139/G06-036 16936788

[B68] Devenish RJ Prescott M Roucou X Nagley P Insights into ATP synthase assembly and function through the molecular genetic manipulation of subunits of the yeast mitochondrial enzyme complex BBA - Bioenergetics 2000 1458 428 442 10.1016/S0005-2728(00)00092-X 10838056

[B69] Pesole G Gissi C De Chirico A Saccone C Nucleotide substitution rate of mammalian mitochondrial genomes J Mol Evol 1999 48 427 434 10.1007/PL00006487 10079281

[B70] Yu Z Wei Z Kong X Shi W Complete mitochondrial DNA sequence of oyster *Crassostrea hongkongensis *-a case of "Tandem duplication-random loss" for genome rearrangement in *Crassostrea *? BMC Genomics 2008 9 477 10.1186/1471-2164-9-477 18847502 PMC2576254

[B71] Okimoto R Macfarlane JL Clary DO Wolstenholme DR The mitochondrial genomes of two nematodes, *Caenorhabditis elegans *and *Ascaris suum* Genetics 1992 130 471 498 1551572 10.1093/genetics/130.3.471 PMC1204866

[B72] Le TH Blair D Agatsuma T Humair PF Campbell NJ Iwagami M Littlewood DT Peacock B Johnston DA Bartley J Rollinson D Herniou EA Zarlenga DS McManus DP Phylogenies inferred from mitochondrial gene orders--A cautionary tale from the parasitic flatworms Mol Biol Evol 2000 17 1123 1125 10889225 10.1093/oxfordjournals.molbev.a026393

[B73] Wolstenholme DR Wolstenholme DR, Jeon KW Animal mitochondrial DNA: structure and evolution Mitochondrial genomes. International Review of Cytology 1992 141 New York: Academic Press 173 216 10.1016/S0074-7696(08)62066-5 1452431

[B74] Reyes A Gissi C Pesole G Saccone C Asymmetrical directional mutation pressure in the mitochondrial genome of mammals Mol Biol Evol 1998 15 957 966 9718723 10.1093/oxfordjournals.molbev.a026011

[B75] Fuller KM Zouros E Dispersed discrete length polymorphism of mitochondrial DNA in the scallop *Placopecten magellanicus *(Gmelin) Curr Genet 1993 23 365 369 10.1007/BF00310901 8096801

[B76] Rigaa A Monnerot M Sellos D Molecular cloning and complete nucleotide sequence of the repeated unit and flanking gene of the scallop *Pecten maximus *mitochondrial DNA: putative replication origin features J Mol Evol 1995 41 189 195 10.1007/BF00170672 7666448

[B77] Carapelli A Comandi S Convey P Nardi F Frati F The complete mitochondrial genome of the Antarctic springtail *Cryptopygus antarcticus *(Hexapoda: Collembola) BMC Genomics 2008 9 315 327 10.1186/1471-2164-9-315 18593463 PMC2483729

[B78] Breton S Beaupre HD Stewart DT Piontkivska H Karmakar M Bogan AE Blier PU Hoeh WR Comparative mitochondrial genomics of freshwater mussels (Bivalvia: Unionoida) with doubly uniparental inheritance of mtDNA: gender-specific open reading frames and putative origins of replication Genetics 2009 183 1575 1589 10.1534/genetics.109.110700 19822725 PMC2787441

[B79] Blier PU Dufresne F Burton RS Natural selection and the evolution of mtDNA-encoded peptides: evidence for intergenomic co-adaptation Trends Genet 2001 17 400 406 10.1016/S0168-9525(01)02338-1 11418221

[B80] Blier PU Breton S Desrosiers V Lemieux H Functional conservatism in mitochondrial evolution: Insight from hybridization of arctic and brook charrs J Exp Zool B Mol Dev Evol 2006 306B 425 432 10.1002/jez.b.21089 16404737

[B81] Hoeh WR Stewart DT Saavedra C Sutherland BW Zouros E Phylogenetic evidence for role-reversals of gender-associated mitochondrial DNA in *Mytilus *(Bivalvia: Mytilidae) Mol Biol Evol 1997 14 959 967 9287429 10.1093/oxfordjournals.molbev.a025839

[B82] Saavedra C Reyero MI Zouros E Male-dependent doubly uniparental inheritance of mitochondrial DNA and female-dependent sex-ratio in the mussel *Mytilus galloprovincialis* Genetics 1997 145 1073 1082 9093859 10.1093/genetics/145.4.1073 PMC1207877

[B83] Stewart DT Breton S Blier PU Hoeh WR Pontarotti P Masculinization events and doubly uniparental inheritance of mitochondrial DNA: A model for understanding the evolutionary dynamics of gender-asssociated mtDNA in mussels Evolutionary Biology from Concept to Application II Berlin: Springer-Verlag in press

[B84] Jha M Côté J Hoeh WR Blier PU Stewart DT Sperm motility in Mytilus edulis in relation to mitochondrial DNA polymorphisms: implications for the evolution of doubly uniparental inheritance in bivalves Evolution 2008 62 99 106 18039328 10.1111/j.1558-5646.2007.00262.x

[B85] Breton S Stewart DT Blier PU Role-reversal of gender-associated mitochondrial DNA affects mitochondrial function in *Mytilus edulis *(Bivalvia: Mytilidae) J Exp Zool B Mol Dev Evol 2009 312B 108 117 10.1002/jez.b.20251 19097171

[B86] Buhay JE Serb JM Dean CR Parham Q Lydeard C Conservation genetics of two endangered unionid bivalve species, *Epioblasma florentina walkeri *and *E. Capsaeformis *(Unionidae: Lampsilini) J Mollus Stud 2002 68 385 391 10.1093/mollus/68.4.385

[B87] Palumbi SR Hillis DM, Moritz C, Mable BK Nucleic Acids II: The Polymerase Chain Reaction Molecular Systematics 1996 Sunderland: Sinauer Associates 205 247

[B88] Wyman SK Jansen RK Boore JL Automatic annotation of organellar genomes with DOGMA Bioinformatics 2004 20 3252 3255 10.1093/bioinformatics/bth352 15180927

[B89] Thompson JD Higgins DJ Gibson TJ CLUSTAL W: improving the sensitivity of progressive multiple sequence alignment through sequence weighting, position-specific gap penalties and weight matrix choice Nucl Acids Res 1994 22 4673 4680 10.1093/nar/22.22.4673 7984417 PMC308517

[B90] Lowe TM Eddy SR tRNAscan-SE: a program for improved detection of transfer RNA genes in genomic sequence Nucl Acids Res 1997 25 955 964 10.1093/nar/25.5.955 9023104 PMC146525

[B91] Laslett D Canback B ARWEN: a program to detect tRNA genes in metazoan mitochondrial nucleotide sequences Bioinformatics 2008 24 172 175 10.1093/bioinformatics/btm573 18033792

[B92] Kumar S Tamura K Jakobsen IB Nei M MEGA2: molecular evolutionary genetics analysis software Bioinformatics 2001 17 1244 1245 10.1093/bioinformatics/17.12.1244 11751241

[B93] Rozas J Sanchez-DelBarrio JC Messeguer X Rozas R DnaSP, DNA polymorphism analyses by the coalescent and other methods Bioinformatics 2003 19 2496 2497 10.1093/bioinformatics/btg359 14668244

[B94] Nei M Gojobori T Simple methods for estimating the numbers of synonymous and nonsynonymous nucleotide substitutions Mol Biol Evol 1986 3 418 426 3444411 10.1093/oxfordjournals.molbev.a040410

[B95] Hassanin A Leger N Deutsch J Evidence for multiple reversals of asymmetric mutational constraints during the evolution of the mitochondrial genome of Metazoa, and consequences for phylogenetic inferences Syst Biol 2005 54 277 298 10.1080/10635150590947843 16021696

[B96] Larkin MA Blackshields G Brown NP Chenna R McGettigan PA McWilliam H Valentin F Wallace IM Wilm A Lopez R Thompson JD Gibson TJ Higgins DG ClustalW and ClustalX version 2 Bioinformatics 2007 23 2947 2948 10.1093/bioinformatics/btm404 17846036

[B97] Subramanian AR Kaufmann M Morgenstern B DIALIGN-TX: greedy and progressive approaches for segment-based multiple sequence alignment Algorithms Mol Biol 2008 3 6 10.1186/1748-7188-3-6 18505568 PMC2430965

[B98] Huelsenbeck JP Ronquist F MRBAYES: Bayesian inference of phylogenetic trees Bioinformatics 2001 17 754 755 10.1093/bioinformatics/17.8.754 11524383

[B99] Ronquist F Huelsenbeck JP MrBayes 3: Bayesian phylogenetic inference under mixed models Bioinformatics 2003 19 1572 1574 10.1093/bioinformatics/btg180 12912839

[B100] Yang Z Nielsen R Goldman N Pedersen AMK Codon-substitution models for heterogeneous selection pressure at amino acid sites Genetics 2000 155 431 449 10790415 10.1093/genetics/155.1.431 PMC1461088

[B101] Ronquist F Huelsenbeck JP Mark P van der MrBayes 3.1 manual 2005 http://mrbayes.csit.fsu.edu/mb3.1_manual.pdf

[B102] Zwickl DJ Genetic algorithm approaches for the phylogenetic analysis of large biological sequence datasets under the maximum likelihood criterion PhD thesis 2006 The University of Texas at Austin

[B103] Felsenstein J Confidence limits on phylogenies: An approach using the bootstrap Evolution 1985 39 783 791 10.2307/2408678 28561359

[B104] Whelan S Goldman N A general empirical model of protein evolution derived from multiple protein families using a maximum likelihood approach Mol Biol Evol 2001 18 691 699 11319253 10.1093/oxfordjournals.molbev.a003851

[B105] Swofford DL PAUP*. Phylogenetic Analysis Using Parsimony (*and Other Methods) 2002 Sunderland: Sinauer Associates http://paup.csit.fsu.edu12504223

[B106] Maddison WP Maddison DR Mesquite: a modular system for evolutionary analysis. Version 2.6 2008 http://mesquiteproject.org

[B107] Maddison DR Maddison WP MacClade 4: Analysis of Parsimony and Character Evolution 2003 4.06 Sunderland: Sinauer Associates

[B108] Pagel M The maximum likelihood approach to reconstructing ancestral character states of discrete characters on phylogenies Syst Biol 1999 48 612 622 10.1080/106351599260184

[B109] Edwards AWF Likelihood 1972 Cambridge: Cambridge University Press

[B110] Fernandez AA Morris MR Sexual selection and trichromatic color vision in primates: Statistical support for the preexisting-bias hypothesis Am Nat 2007 170 10 20 10.1086/518566 17853988

[B111] Murphy NP Carey D Castro LR Dowton M Austin AD Phylogeny of the platygastroid wasps (Hymenoptera) based on sequences from the 18S rRNA, 28S rRNA and cytochrome oxidase I genes: implications for the evolution of the ovipositor system and host relationships Biol J Linnean 2007 91 653 669 10.1111/j.1095-8312.2007.00825.x

[B112] Koepfli KP Deere KA Slater GJ Begg C Begg K Grassman L Lucherini M Veron G Wayne RK Multigene phylogeny of the Mustelidae: Resolving relationships, tempo and biogeographic history of a mammalian adaptive radiation BMC Biol 2008 6 10 10.1186/1741-7007-6-10 18275614 PMC2276185

[B113] Conant GC Wolfe KH GenomeVx: simple web-based creation of editable circular chromosome maps Bioinformatics 2008 24 861 862 10.1093/bioinformatics/btm598 18227121

