# Supplementary material for: Mitochondrial phylogenomics of the Bivalvia (Mollusca): searching for the origin and mitogenomic correlates of doubly uniparental inheritance of mtDNA
Source: BMC Evol Biol. 2010 Feb 18;10:50. doi: 10.1186/1471-2148-10-50 (PMC2834691; doi:10.1186/1471-2148-10-50)
Supplement: Additional file 1 — Supplemental figures. Figure S1. Inferred secondary structures of the 22 mitochondrial tRNAs from F Venustaconcha ellipsiformis, shown in the order they occur in the genome, beginning with trnH. Amino acid identities are given above each sequence. Figure S2. Inferred secondary structures of the 22 mitochondrial tRNAs from M Venustaconcha ellipsiformis, shown in the order they occur in the genome, beginning with trnA. Amino acid identities are given above each sequence. Figure S3. Inferred secondary structures of the 22 mitochondrial tRNAs from F Pyganodon grandis, shown in the order they occur in the genome, beginning with trnH. Amino acid identities are given above each sequence. Figure S4. Inferred secondary structures of the 22 mitochondrial tRNAs from M Pyganodon grandis, shown in the order they occur in the genome, beginning with trnA. Amino acid identities are given above each sequence. Figure S5. Inferred secondary structures of the 22 mitochondrial tRNAs from F Inversidens japanensis, shown in the order they occur in the genome, beginning with trnH. Amino acid identities are given above each sequence. Figure S6. Inferred secondary structures of the 22 mitochondrial tRNAs from M Inversidens japanensis, shown in the order they occur in the genome, beginning with trnA. Amino acid identities are given above each sequence. Figure S7. Inferred secondary structures of the 22 mitochondrial tRNAs from F Quadrula quadrula, shown in the order they occur in the genome, beginning with trnH. Amino acid identities are given above each sequence. Figure S8. Inferred secondary structures of the 22 mitochondrial tRNAs from M Quadrula quadrula, shown in the order they occur in the genome, beginning with trnA. Amino acid identities are given above each sequence. [file 1471-2148-10-50-S1.PDF]

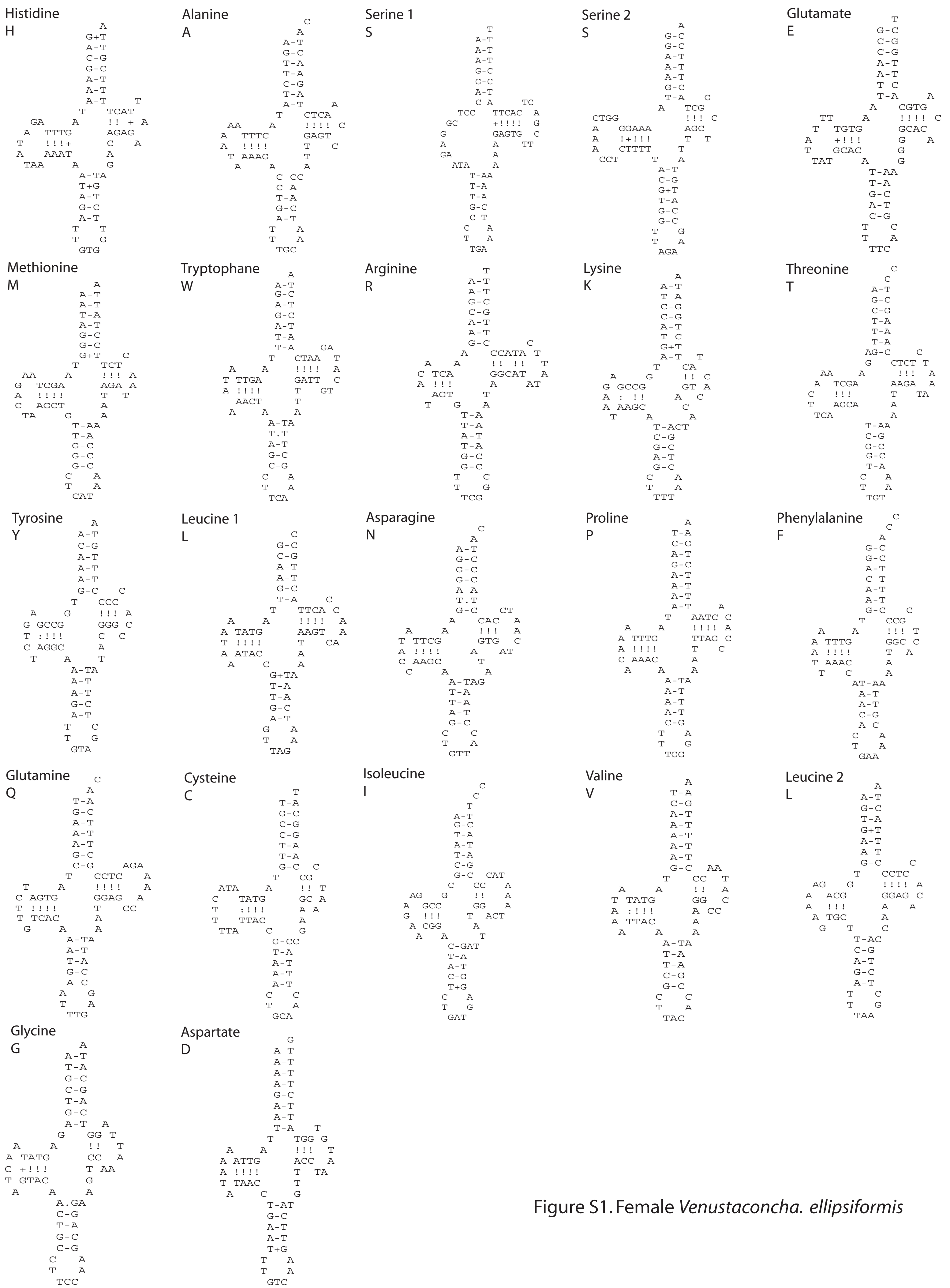

Figure S1. Female *Venustaconcha. ellipsiformis*

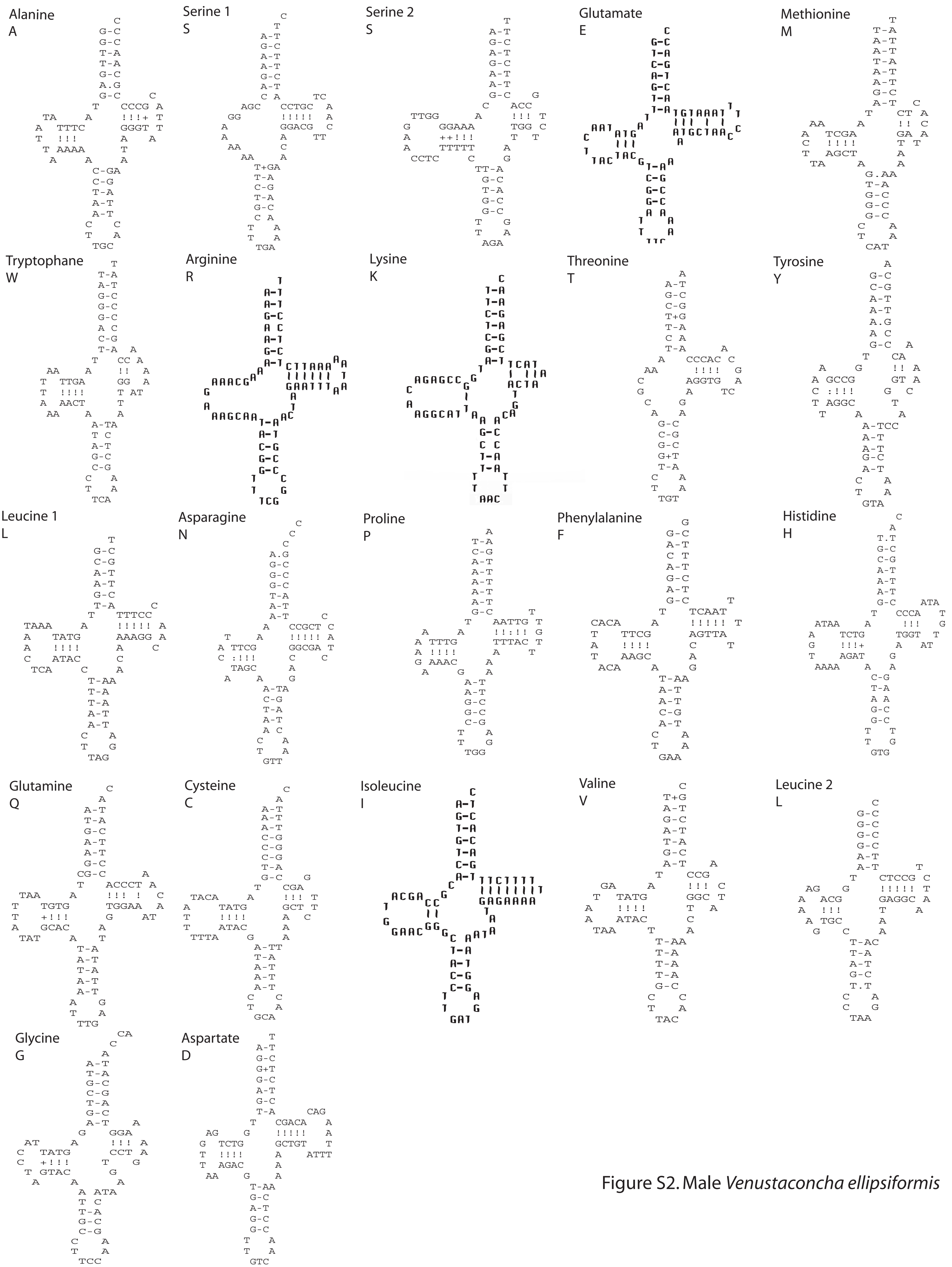

Figure S2. Male *Venustaconcha ellipsiformis*

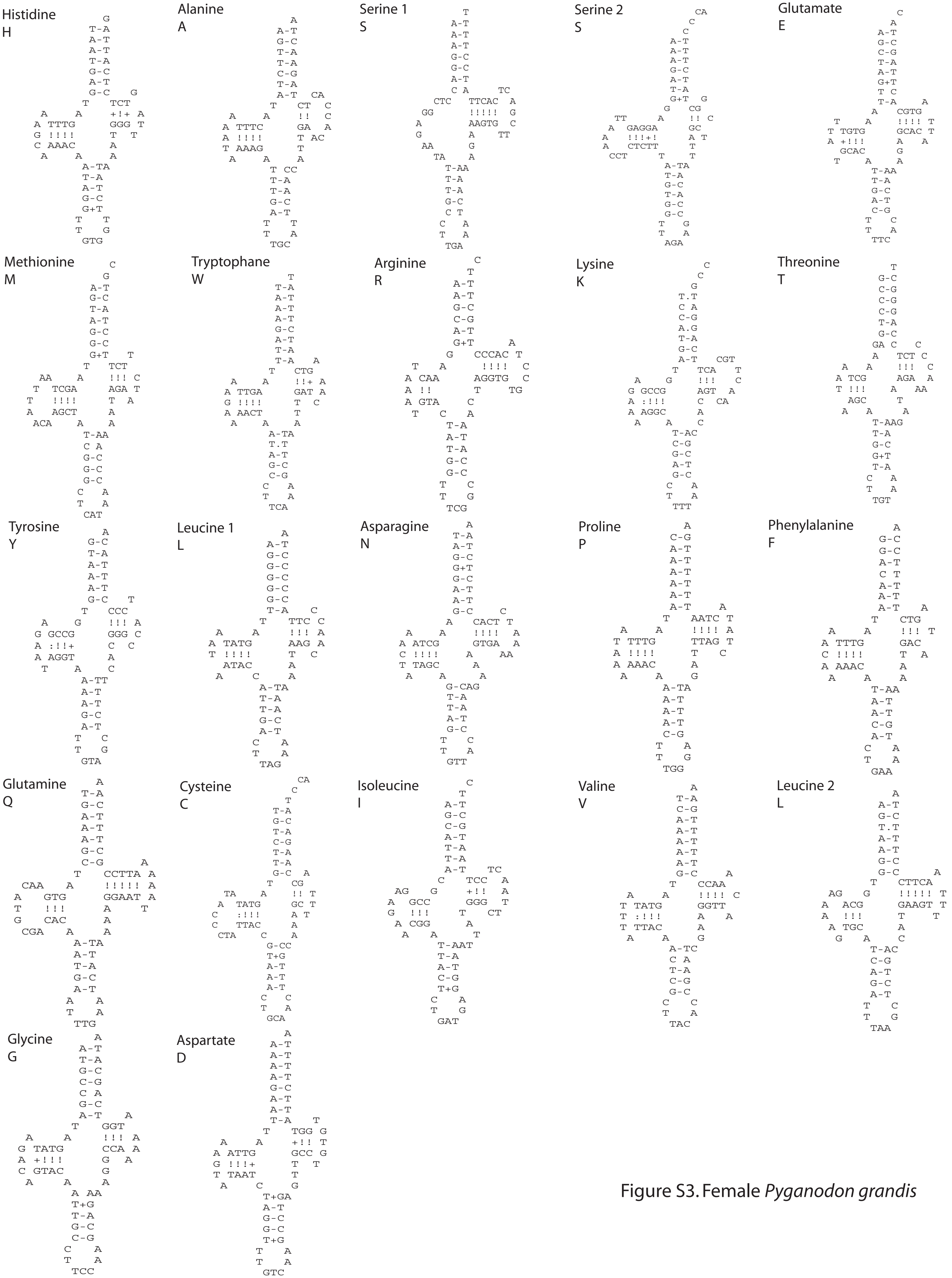

Figure S3. Female *Pyganodon grandis*

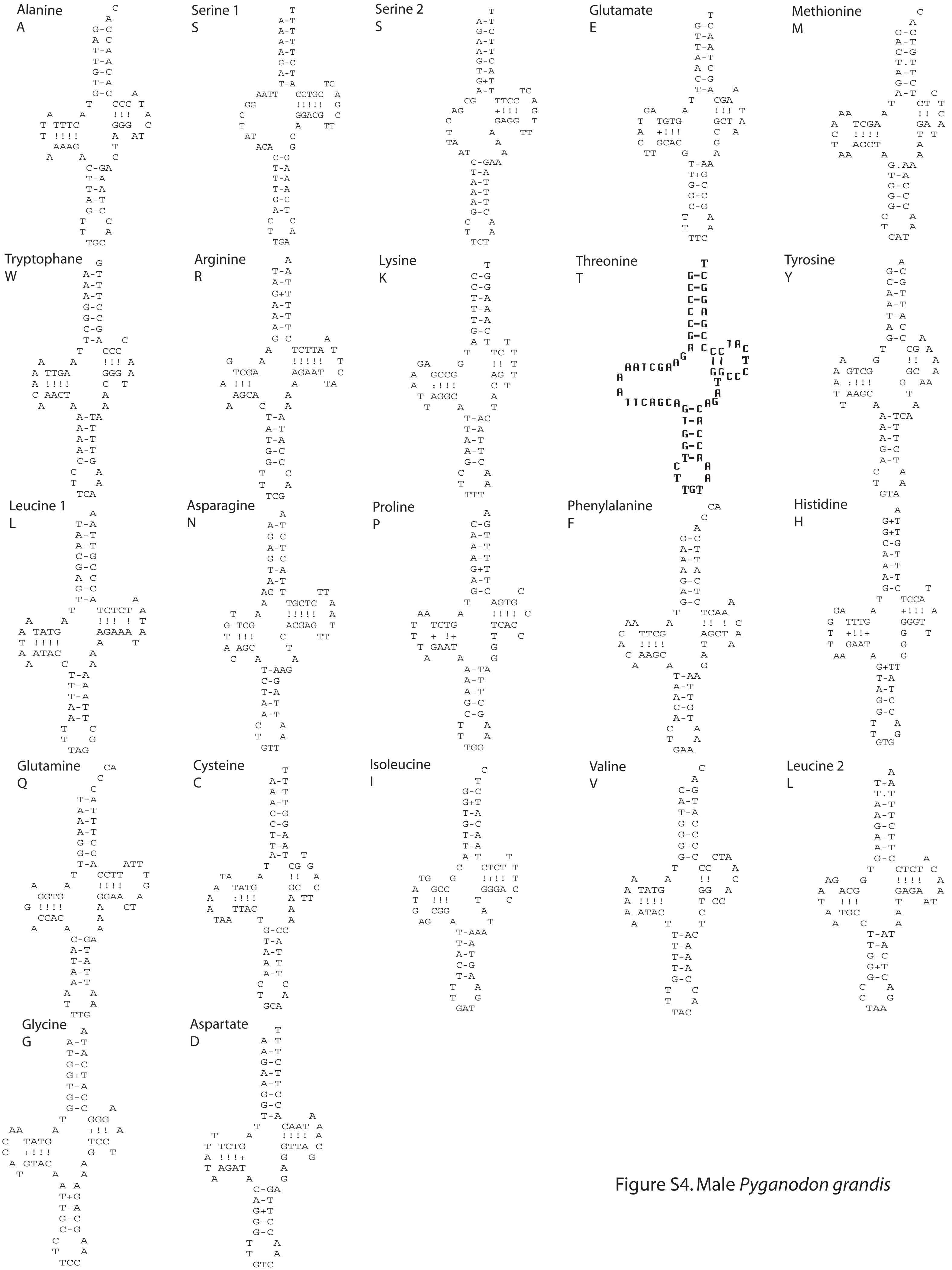

Figure S4. Male *Pyganodon grandis*

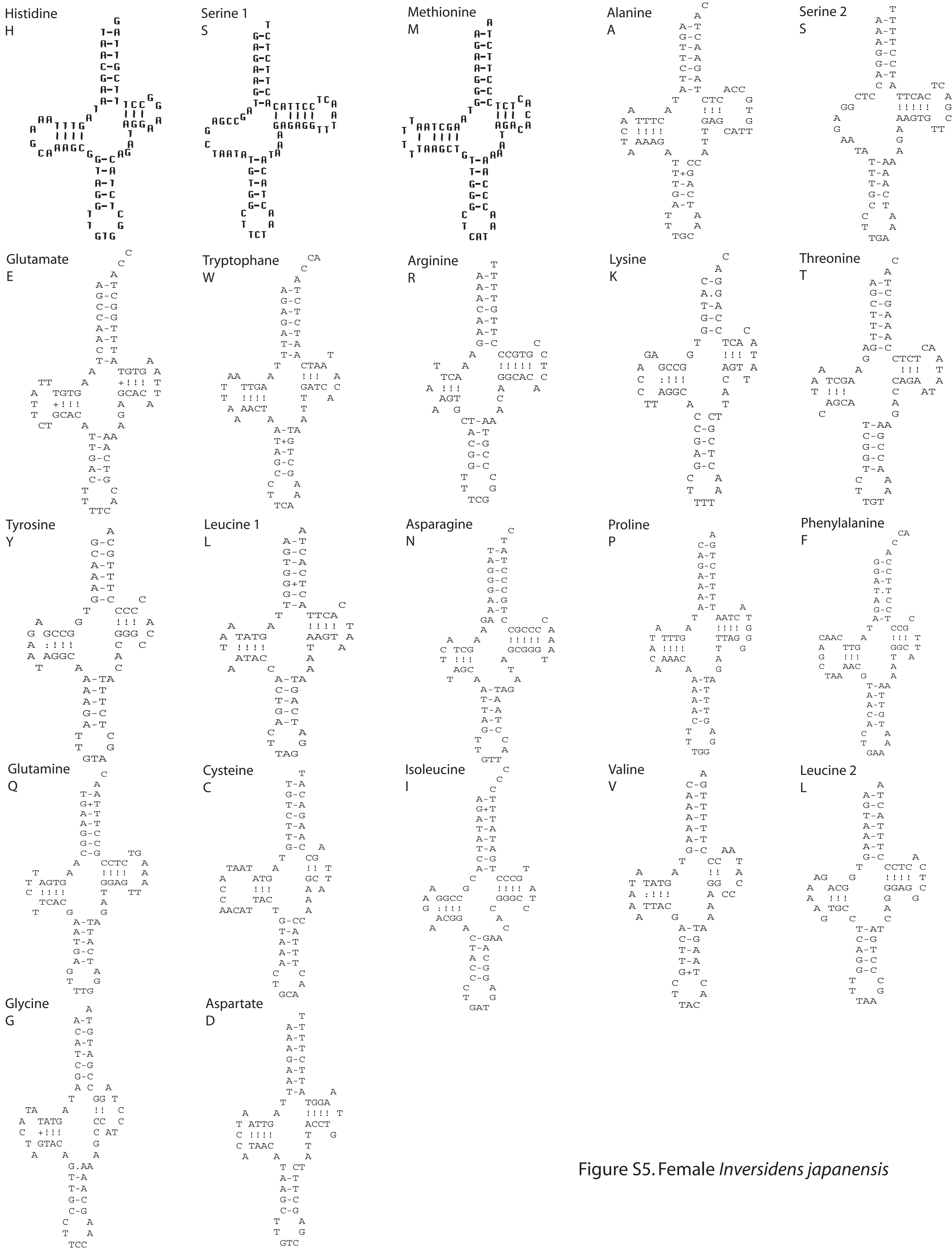

Figure S5. Female *Inversidens japanensis*

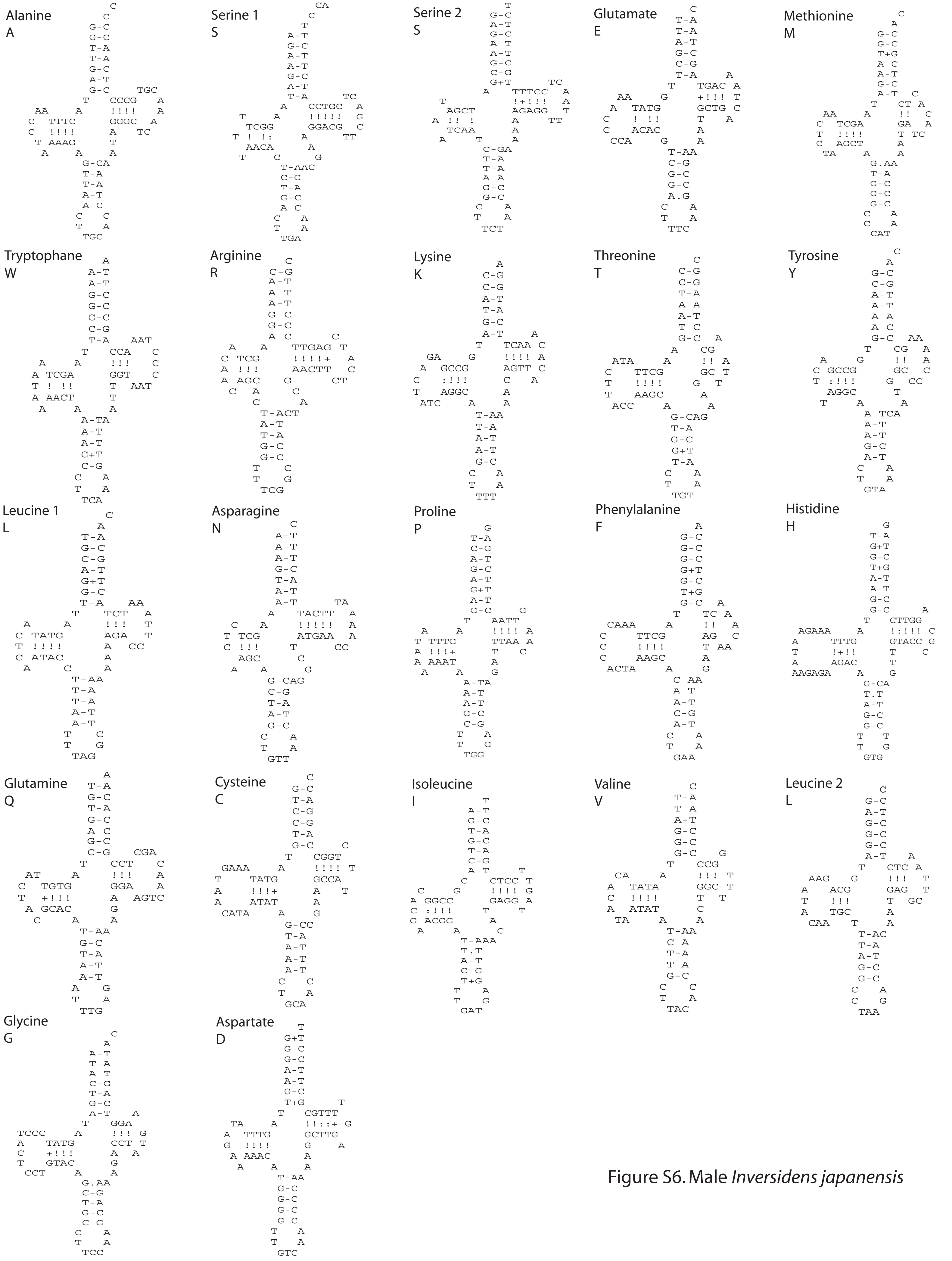

Figure S6. Male *Inversidens japanensis*

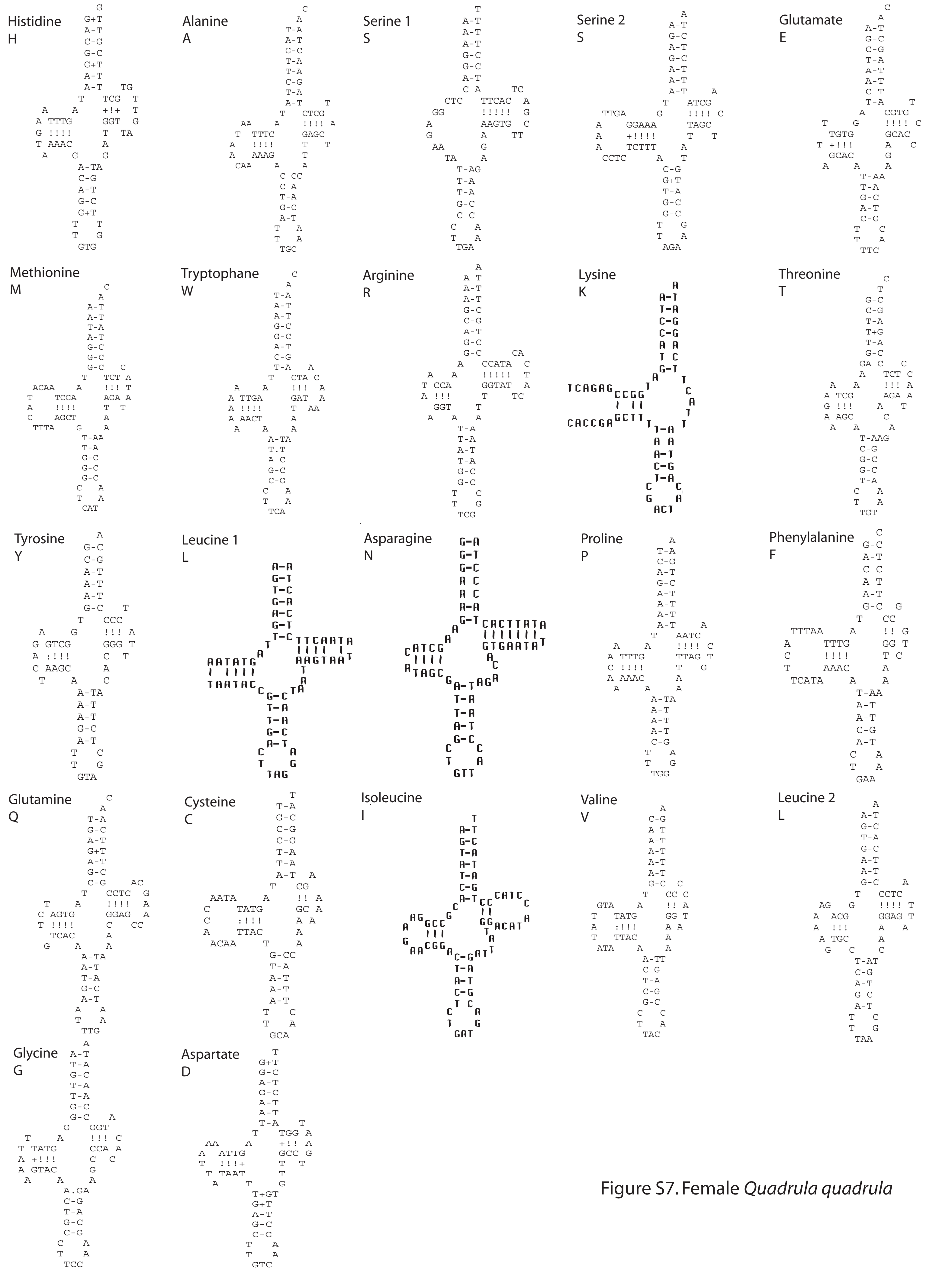

Figure S7. Female *Quadrula quadrula*

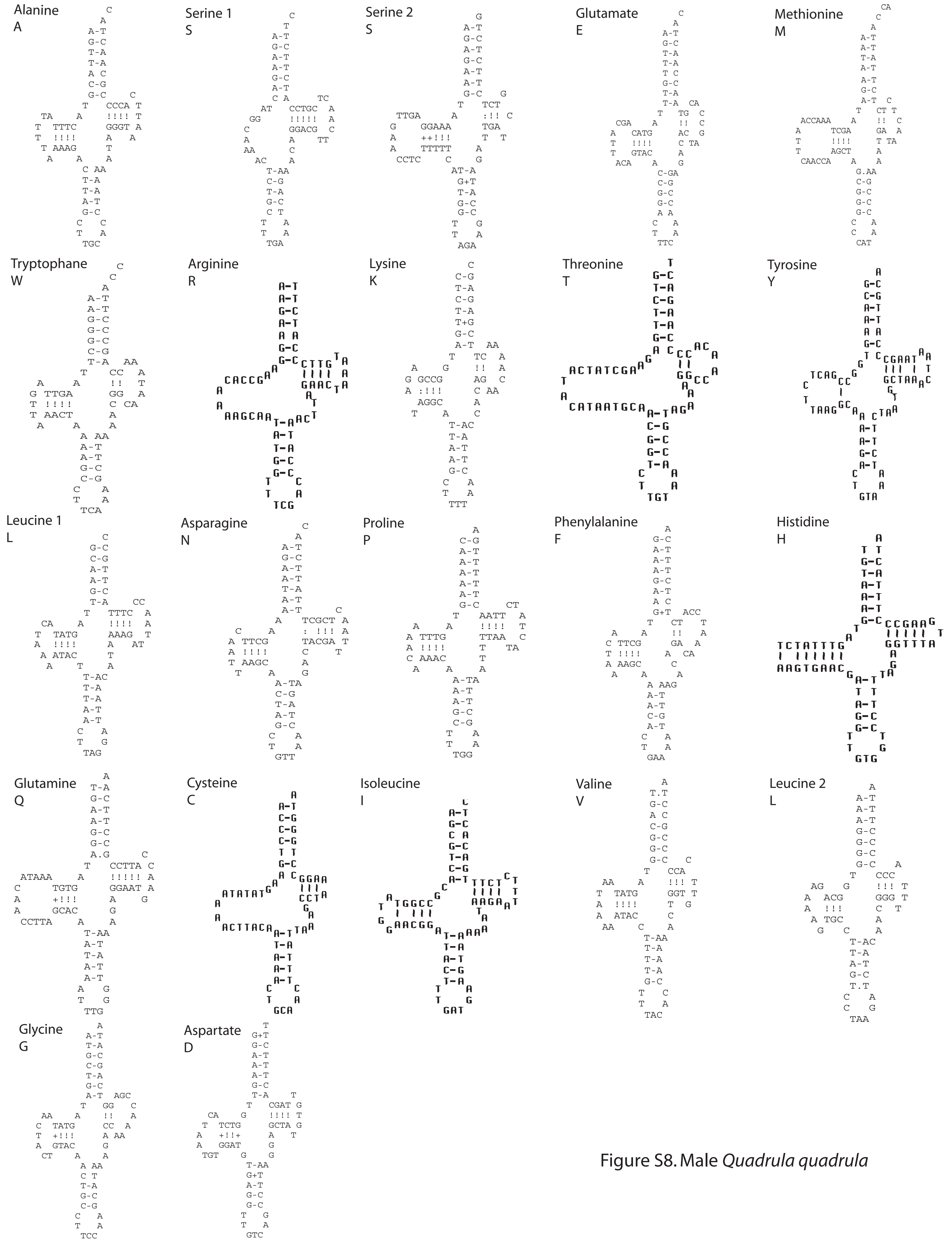

Figure S8. Male *Quadrula quadrula*
