# Supplementary material for: Mitochondrial phylogenomics of the Bivalvia (Mollusca): searching for the origin and mitogenomic correlates of doubly uniparental inheritance of mtDNA
Source: BMC Evol Biol. 2010 Feb 18;10:50. doi: 10.1186/1471-2148-10-50 (PMC2834691; doi:10.1186/1471-2148-10-50)
Supplement: Additional file 2 — Table S1. Codon usage in the female- and male-transmitted mitochondrial genomes of Venustaconcha ellipsiformis, Pyganodon grandis, Inversidens japanensis and Quadrula quadrula. Table of the codon usage in the female- and male-transmitted mitochondrial genomes of Venustaconcha ellipsiformis, Pyganodon grandis, Inversidens japanensis and Quadrula quadrula. [file 1471-2148-10-50-S2.DOC]

Supplementary Table S1

Codon usage in the female- and male-transmitted mitochondrial genomes of *V. ellipsiformis*, *P. grandis*, *I. japanenesis* and *Q. quadrula*.

|  |  | ***V. ellipsiformis***  **Female** | | ***V. ellipsiformis***  **Male** | | ***P. grandis***  **Female** | | ***P. grandis***  **Male** | | | ***I. japanensis***  **Female** | | | ***I. japanensis***  **Male** | | | ***Q. quadrula***  **Female** | | | ***Q. quadrula***  **Male** | | |
| --- | --- | --- | --- | --- | --- | --- | --- | --- | --- | --- | --- | --- | --- | --- | --- | --- | --- | --- | --- | --- | --- | --- |
| **AA** | **Codon** | **N** | **%** | **N** | **%** | **N** | **%** | **N** | **%** | **N** | | **%** | **N** | | **%** | **N** | | **%** | **N** | | **%** |  |
| Phe (F) | TTT | 278 | 7.46 | 274 | 7.06 | 255 | 6.85 | 233 | 6.32 | 196 | | 5.00 | 259 | | 6.83 | 283 | | 7.65 | 262 | | 6.71 |  |
|  | TTC | 33 | 0.89 | 47 | 1.21 | 45 | 1.21 | 59 | 1.60 | 61 | | 1.56 | 57 | | 1.50 | 47 | | 1.27 | 42 | | 1.08 |  |
| Leu (L1) | TTA | 187 | 5.02 | 157 | 4.05 | 231 | 6.20 | 137 | 3.71 | 86 | | 2.19 | 217 | | 5.72 | 168 | | 4.54 | 157 | | 4.02 |  |
|  | TTG | 184 | 4.94 | 195 | 5.02 | 128 | 3.44 | 188 | 5.10 | 144 | | 3.68 | 122 | | 3.22 | 205 | | 5.54 | 191 | | 4.89 |  |
| Ser (S2) | TCT | 117 | 3.14 | 128 | 3.30 | 121 | 3.25 | 100 | 2.71 | 95 | | 2.42 | 119 | | 3.14 | 118 | | 3.19 | 104 | | 2.67 |  |
|  | TCC | 27 | 0.72 | 18 | 0.46 | 22 | 0.59 | 40 | 1.08 | 37 | | 0.94 | 20 | | 0.53 | 17 | | 0.46 | 21 | | 0.54 |  |
|  | TCA | 45 | 1.21 | 37 | 0.95 | 43 | 1.15 | 31 | 0.84 | 38 | | 0.97 | 37 | | 0.98 | 46 | | 1.24 | 47 | | 1.20 |  |
|  | TCG | 12 | 0.32 | 24 | 0.62 | 13 | 0.35 | 26 | 0.70 | 23 | | 0.59 | 17 | | 0.45 | 17 | | 0.46 | 15 | | 0.38 |  |
| Tyr (Y) | TAT | 90 | 2.42 | 106 | 2.73 | 79 | 2.12 | 81 | 2.20 | 86 | | 2.19 | 90 | | 2.37 | 91 | | 2.46 | 102 | | 2.61 |  |
|  | TAC | 39 | 1.05 | 52 | 1.34 | 51 | 1.37 | 42 | 1.14 | 60 | | 1.53 | 48 | | 1.27 | 37 | | 1.00 | 41 | | 1.05 |  |
| TERM | TAA | 5 | 0.13 | 9 | 0.23 | 8 | 0.21 | 7 | 0.19 | 75 | | 1.91 | 8 | | 0.21 | 9 | | 0.24 | 6 | | 0.15 |  |
|  | TAG | 7 | 0.19 | 3 | 0.08 | 5 | 0.13 | 5 | 0.14 | 52 | | 1.33 | 2 | | 0.05 | 4 | | 0.11 | 4 | | 0.10 |  |
| Cys (C) | TGT | 50 | 1.34 | 85 | 2.19 | 41 | 1.10 | 41 | 1.11 | 69 | | 1.76 | 69 | | 1.82 | 50 | | 1.35 | 87 | | 2.23 |  |
|  | TGC | 11 | 0.30 | 8 | 0.21 | 14 | 0.38 | 18 | 0.49 | 30 | | 0.77 | 25 | | 0.66 | 10 | | 0.27 | 14 | | 0.36 |  |
| Trp (W) | TGA | 39 | 1.05 | 43 | 1.11 | 57 | 1.53 | 38 | 1.03 | 55 | | 1.40 | 68 | | 1.79 | 44 | | 1.19 | 52 | | 1.33 |  |
|  | TGG | 75 | 2.01 | 69 | 1.78 | 58 | 1.56 | 68 | 1.84 | 108 | | 2.76 | 43 | | 1.13 | 71 | | 1.92 | 64 | | 1.64 |  |
| Leu (L2) | CTT | 77 | 2.07 | 78 | 2.01 | 76 | 2.04 | 70 | 1.90 | 103 | | 2.63 | 99 | | 2.61 | 76 | | 2.05 | 99 | | 2.54 |  |
|  | CTC | 26 | 0.70 | 17 | 0.44 | 24 | 0.64 | 45 | 1.22 | 48 | | 1.23 | 19 | | 0.50 | 23 | | 0.62 | 31 | | 0.79 |  |
|  | CTA | 72 | 1.93 | 83 | 2.14 | 88 | 2.36 | 71 | 1.92 | 80 | | 2.04 | 83 | | 2.19 | 69 | | 1.87 | 91 | | 2.33 |  |
|  | CTG | 20 | 0.54 | 19 | 0.49 | 25 | 0.67 | 50 | 1.36 | 49 | | 1.25 | 29 | | 0.76 | 16 | | 0.43 | 18 | | 0.46 |  |
| Pro (P) | CCT | 56 | 1.50 | 54 | 1.39 | 66 | 1.77 | 49 | 1.33 | 59 | | 1.51 | 65 | | 1.71 | 55 | | 1.49 | 51 | | 1.31 |  |
|  | CCC | 18 | 0.48 | 9 | 0.23 | 17 | 0.46 | 29 | 0.79 | 78 | | 1.99 | 14 | | 0.37 | 4 | | 0.11 | 6 | | 0.15 |  |
|  | CCA | 48 | 1.29 | 57 | 1.47 | 38 | 1.02 | 42 | 1.14 | 63 | | 1.61 | 40 | | 1.05 | 61 | | 1.65 | 53 | | 1.36 |  |
|  | CCG | 18 | 0.48 | 9 | 0.23 | 13 | 0.35 | 26 | 0.70 | 31 | | 0.79 | 8 | | 0.21 | 18 | | 0.49 | 14 | | 0.36 |  |
| His (H) | CAT | 62 | 1.66 | 49 | 1.26 | 56 | 1.50 | 45 | 1.22 | 49 | | 1.25 | 58 | | 1.53 | 54 | | 1.46 | 60 | | 1.54 |  |
|  | CAC | 16 | 0.43 | 31 | 0.80 | 20 | 0.54 | 31 | 0.84 | 61 | | 1.56 | 19 | | 0.50 | 19 | | 0.51 | 25 | | 0.64 |  |
| Gln (Q) | CAA | 38 | 1.02 | 40 | 1.03 | 41 | 1.10 | 29 | 0.79 | 93 | | 2.37 | 44 | | 1.16 | 39 | | 1.05 | 43 | | 1.10 |  |
|  | CAG | 24 | 0.64 | 22 | 0.57 | 20 | 0.54 | 32 | 0.87 | 38 | | 0.97 | 23 | | 0.61 | 23 | | 0.62 | 24 | | 0.62 |  |
| Arg (R) | CGT | 12 | 0.32 | 30 | 0.77 | 13 | 0.35 | 18 | 0.49 | 34 | | 0.87 | 21 | | 0.55 | 18 | | 0.49 | 27 | | 0.69 |  |
|  | CGC | 6 | 0.16 | 3 | 0.08 | 5 | 0.13 | 7 | 0.19 | 10 | | 0.26 | 2 | | 0.05 | 3 | | 0.08 | 6 | | 0.15 |  |
|  | CGA | 25 | 0.67 | 16 | 0.41 | 37 | 0.99 | 24 | 0.65 | 16 | | 0.41 | 25 | | 0.66 | 21 | | 0.57 | 19 | | 0.49 |  |
|  | CGG | 16 | 0.43 | 12 | 0.31 | 6 | 0.16 | 16 | 0.43 | 21 | | 0.54 | 9 | | 0.24 | 21 | | 0.57 | 10 | | 0.26 |  |
| Ile (I) | ATT | 197 | 5.29 | 185 | 4.77 | 222 | 5.96 | 148 | 4.01 | 94 | | 2.40 | 194 | | 5.11 | 188 | | 5.08 | 140 | | 3.59 |  |
|  | ATC | 49 | 1.32 | 53 | 1.37 | 52 | 1.40 | 56 | 1.52 | 58 | | 1.48 | 49 | | 1.29 | 54 | | 1.46 | 70 | | 1.79 |  |
| Met (M) | ATA | 108 | 2.90 | 117 | 3.01 | 118 | 3.17 | 79 | 2.14 | 74 | | 1.89 | 140 | | 3.69 | 91 | | 2.46 | 117 | | 3.00 |  |
|  | ATG | 65 | 1.74 | 91 | 2.34 | 61 | 1.64 | 85 | 2.30 | 74 | | 1.89 | 72 | | 1.90 | 74 | | 2.00 | 97 | | 2.49 |  |
| Thr (T) | ACT | 73 | 1.96 | 86 | 2.22 | 74 | 1.99 | 71 | 1.92 | 65 | | 1.66 | 83 | | 2.19 | 61 | | 1.65 | 81 | | 2.08 |  |
|  | ACC | 42 | 1.13 | 27 | 0.70 | 24 | 0.64 | 49 | 1.33 | 58 | | 1.48 | 26 | | 0.69 | 39 | | 1.05 | 36 | | 0.92 |  |
|  | ACA | 50 | 1.34 | 65 | 1.67 | 62 | 1.66 | 39 | 1.06 | 62 | | 1.58 | 59 | | 1.56 | 53 | | 1.43 | 65 | | 1.67 |  |
|  | ACG | 10 | 0.27 | 12 | 0.31 | 11 | 0.30 | 21 | 0.57 | 15 | | 0.38 | 11 | | 0.29 | 14 | | 0.38 | 18 | | 0.46 |  |
| Asn (N) | AAT | 57 | 1.53 | 67 | 1.73 | 60 | 1.61 | 52 | 1.41 | 65 | | 1.66 | 70 | | 1.85 | 54 | | 1.46 | 54 | | 1.38 |  |
|  | AAC | 30 | 0.81 | 44 | 1.13 | 41 | 1.10 | 41 | 1.11 | 80 | | 2.04 | 41 | | 1.08 | 38 | | 1.03 | 41 | | 1.05 |  |
| Lys (K) | AAA | 50 | 1.34 | 48 | 1.24 | 58 | 1.56 | 43 | 1.17 | 75 | | 1.91 | 64 | | 1.69 | 42 | | 1.14 | 47 | | 1.20 |  |
|  | AAG | 42 | 1.13 | 49 | 1.26 | 32 | 0.86 | 35 | 0.95 | 48 | | 1.23 | 36 | | 0.95 | 39 | | 1.05 | 48 | | 1.23 |  |
| Ser (S1) | AGT | 56 | 1.50 | 71 | 1.83 | 61 | 1.64 | 35 | 0.95 | 42 | | 1.07 | 54 | | 1.42 | 60 | | 1.62 | 74 | | 1.90 |  |
|  | AGC | 12 | 0.32 | 12 | 0.31 | 20 | 0.54 | 23 | 0.62 | 49 | | 1.25 | 31 | | 0.82 | 15 | | 0.41 | 21 | | 0.54 |  |
|  | AGA | 36 | 0.97 | 54 | 1.39 | 60 | 1.61 | 26 | 0.70 | 39 | | 1.00 | 84 | | 2.21 | 36 | | 0.97 | 40 | | 1.03 |  |
|  | AGG | 51 | 1.37 | 59 | 1.52 | 49 | 1.32 | 66 | 1.79 | 43 | | 1.10 | 66 | | 1.74 | 49 | | 1.32 | 62 | | 1.59 |  |
| Val (V) | GTT | 190 | 5.10 | 224 | 5.77 | 148 | 3.97 | 170 | 4.61 | 136 | | 3.47 | 139 | | 3.66 | 186 | | 5.03 | 226 | | 5.79 |  |
|  | GTC | 16 | 0.43 | 20 | 0.52 | 27 | 0.72 | 41 | 1.11 | 26 | | 0.66 | 20 | | 0.53 | 20 | | 0.54 | 20 | | 0.51 |  |
|  | GTA | 73 | 1.96 | 83 | 2.14 | 96 | 2.58 | 64 | 1.73 | 58 | | 1.48 | 121 | | 3.19 | 62 | | 1.68 | 88 | | 2.26 |  |
|  | GTG | 91 | 2.44 | 104 | 2.68 | 68 | 1.83 | 110 | 2.98 | 97 | | 2.48 | 58 | | 1.53 | 100 | | 2.70 | 107 | | 2.74 |  |
| Ala (A) | GCT | 106 | 2.85 | 84 | 2.16 | 113 | 3.03 | 111 | 3.01 | 63 | | 1.61 | 85 | | 2.24 | 131 | | 3.54 | 89 | | 2.28 |  |
|  | GCC | 35 | 0.94 | 14 | 0.36 | 33 | 0.89 | 61 | 1.65 | 44 | | 1.12 | 26 | | 0.69 | 26 | | 0.70 | 33 | | 0.85 |  |
|  | GCA | 47 | 1.26 | 41 | 1.06 | 52 | 1.40 | 38 | 1.03 | 44 | | 1.12 | 62 | | 1.63 | 42 | | 1.14 | 49 | | 1.26 |  |
|  | GCG | 19 | 0.51 | 13 | 0.33 | 12 | 0.32 | 32 | 0.87 | 21 | | 0.54 | 20 | | 0.53 | 13 | | 0.35 | 9 | | 0.23 |  |
| Asp (D) | GAT | 58 | 1.56 | 70 | 1.80 | 49 | 1.32 | 52 | 1.41 | 43 | | 1.10 | 52 | | 1.37 | 58 | | 1.57 | 64 | | 1.64 |  |
|  | GAC | 12 | 0.32 | 13 | 0.33 | 15 | 0.40 | 16 | 0.43 | 20 | | 0.51 | 15 | | 0.40 | 11 | | 0.30 | 16 | | 0.41 |  |
| Glu (E) | GAA | 30 | 0.81 | 35 | 0.90 | 51 | 1.37 | 23 | 0.62 | 41 | | 1.05 | 57 | | 1.50 | 38 | | 1.03 | 30 | | 0.77 |  |
|  | GAG | 52 | 1.40 | 52 | 1.34 | 44 | 1.18 | 56 | 1.52 | 48 | | 1.23 | 46 | | 1.21 | 50 | | 1.35 | 55 | | 1.41 |  |
| Gly (G) | GGT | 119 | 3.19 | 122 | 3.14 | 101 | 2.71 | 89 | 2.41 | 88 | | 2.25 | 68 | | 1.79 | 116 | | 3.14 | 117 | | 3.00 |  |
|  | GGC | 25 | 0.67 | 14 | 0.36 | 23 | 0.62 | 49 | 1.33 | 41 | | 1.05 | 18 | | 0.47 | 23 | | 0.62 | 12 | | 0.31 |  |
|  | GGA | 51 | 1.37 | 60 | 1.55 | 68 | 1.83 | 47 | 1.27 | 33 | | 0.84 | 92 | | 2.43 | 62 | | 1.68 | 54 | | 1.38 |  |
|  | GGG | 140 | 3.76 | 108 | 2.78 | 104 | 2.79 | 163 | 4.42 | 126 | | 3.22 | 75 | | 1.98 | 117 | | 3.16 | 136 | | 3.49 |  |
